# Evaluating a Large Language Model’s Ability to Synthesize a Health Science Master’s Thesis: Case Study

**DOI:** 10.2196/73248

**Published:** 2025-07-03

**Authors:** Pål Joranger, Sara Rivenes Lafontan, Asgeir Brevik

**Affiliations:** 1 Department of Nursing and Health Promotion, Faculty of Health Sciences, OsloMet – Oslo Metropolitan University, P.O. Box 4 St. Olavs plass, Oslo, N-0130, Norway, 47 67236520

**Keywords:** master’s thesis, large language model, LLM, ChatGPT, health science, qualitative, quantitative

## Abstract

**Background:**

Large language models (LLMs) can aid students in mastering a new topic fast, but for the educational institutions responsible for assessing and grading the academic level of students, it can be difficult to discern whether a text has originated from a student’s own cognition or has been synthesized by an LLM. Universities have traditionally relied on a submitted written thesis as proof of higher-level learning, on which to grant grades and diplomas. But what happens when LLMs are able to mimic the academic writing of subject matter experts? This is now a real dilemma. The ubiquitous availability of LLMs challenges trust in the master’s thesis as evidence of subject matter comprehension and academic competencies.

**Objective:**

In this study, we aimed to assess the quality of rapid machine-generated papers against the standards of the health science master’s program we are currently affiliated with.

**Methods:**

In an exploratory case study, we used ChatGPT (OpenAI) to generate 2 research papers as conceivable student submissions for master’s thesis graduation from a health science master’s program. One paper simulated a qualitative health science research project and another simulated a quantitative health science research project.

**Results:**

Using a stepwise approach, we prompted ChatGPT to (1) synthesize 2 credible datasets, and (2) generate 2 papers, that—in our judgment—would have been able to pass as credible medium-quality graduation research papers at the health science master’s program the authors are currently affiliated with. It took 2.5 hours of iterative dialogue with ChatGPT to develop the qualitative paper and 3.5 hours to develop the quantitative paper. Making the synthetic datasets that served as a starting point for our ChatGPT-driven paper development took 1.5 and 16 hours for the qualitative and quantitative datasets, respectively. This included learning and prompt optimization, and for the quantitative dataset, it included the time it took to create tables, estimate relevant bivariate correlation coefficients, and prepare these coefficients to be read by ChatGPT.

**Conclusions:**

Our demonstration highlights the ease with which an LLM can synthesize research data, conduct scientific analyses, and produce credible research papers required for graduation from a master’s program. A clear and well-written master’s thesis, citing subject matter authorities and true to the expectations for academic writing, can no longer be regarded as solid proof of either extensive study or subject matter mastery. To uphold the integrity of academic standards and the value of university diplomas, we recommend that master’s programs prioritize oral examinations and school exams. This shift is now crucial to ensure a fair and rigorous assessment of higher-order learning and abilities at the master’s level.

## Introduction

Students represent a group that benefits from the use of large language models (LLMs). By using LLMs, students can gain knowledge on virtually any topic as well as edit or create scholarly text instantaneously [1-3]. In a survey from 2023, it was documented that 89% of college students in America used ChatGPT to complete their assignments, and 53% used the tool for writing papers [4]. Another report documents that LLMs perform on par with or better than university students across 32 university courses [5]. Although potentially beneficial for learning, the ubiquitous availability and use of LLMs challenge traditional methods of measuring the quality of students’ academic performance. To obtain a master’s degree, a written thesis is traditionally required. If theses, independent student research projects, or written home examinations can now be easily completed using LLMs, their role as a robust demonstration of higher-level abilities and learning might not be justified going forward.

Despite their already powerful capabilities, LLMs are still relatively new tools with major improvements yet to emerge, making them even more powerful in the future, autonomous executing AI agents being an example [3,6]. It may be argued that many corners of academia are already struggling to keep up with the pace of AI development [7], and more powerful tools can give students an even greater advantage against plagiarism detection attempts. While plagiarism detection tools are widely used at universities, it has been observed that traditional plagiarism detection tools may struggle to detect AI-generated text [8]. This raises concerns that faculty staff could be left with the unappealing task of grading a large number of almost similar student projects written largely by LLMs, falsely believing that they are assessing the student’s academic ability. The value and status of university diplomas could be threatened by the widespread adoption of LLMs.

As health science lecturers and master’s thesis censors at a Norwegian university, we believe there is an urgent need to acknowledge how much of a typical master’s thesis can now be written by LLMs and what this means for assessing students’ abilities in higher education. We were curious to test the ability of one of the most accessible LLMs to assist budding master’s theses’ authors in completing their graduation theses. Hence, we set out to briefly test the power of ChatGPT-4o to assist in the professional analysis and presentation of health science data, both in the realm of qualitative and quantitative data analysis, as well as the writing of an academic paper based on the results. Our objective was to assess the quality of machine-generated papers against the standards of the health science master’s program we are currently affiliated with.

## Methods

### Study Design

In an exploratory case study, we asked ChatGPT (ChatGPT-4o and ChatGPT-o1) to synthesize two datasets: one qualitative and one quantitative, inspired by existing real-world datasets the authors had previously analyzed. The generation of synthetic datasets and subsequent data analysis took place in October and November of 2024.

Two prerequisites were kept during the development of the papers: (1) the process should be feasible for an average student who had completed the methodology courses in our local master’s program, and (2) the process should be possible to complete fast, that is, during a long work session, guided entirely by chatbot dialogue.

### Generation of Qualitative Data

We started by outlining the study aim and instructed ChatGPT-4o mini to prepare interview transcripts of 7 students with different levels of education, age, and gender. We also prompted ChatGPT to develop an interview guide (Multimedia Appendix 1). The first transcripts generated by ChatGTP4o mini were quite short, hence, in a follow-up prompt to ensure that each interview reflected 30‐45 minutes of conversation, we requested interview transcripts that included longer and more detailed responses detailing specific events relevant to the study aim, including respondents’ experiences and feelings. We also prompted for the artificial interviewer to ask follow-up questions, emulating a natural conversation. We prompted 1 interview transcript at a time to ensure that these aspects were included in each transcript. This resulted in 7 transcripts that were considered suitable for further analysis. The qualitative dataset is shown in Multimedia Appendix 2.

### Generation of Quantitative Dataset

For the quantitative data, we used ChatGPT-o1 to develop a script that was run in ChatGPT-4o to produce the data matrix. To work with meaningful associations that resembled what could be found in international literature, we had to provide ChatGPT-o1 with information about how our synthetic dataset’s 10 variables correlated with each other in pairs. To avoid inconsistencies in how the variables correlated as an overall system, we relied on a correlation matrix from real data for similar variables. All the correlation coefficients given to ChatGPT-o1 were discretionarily slightly changed (usually increased slightly) before admission, but without changing the direction (positive or negative) of the correlation. The quantitative dataset is shown in Multimedia Appendix 3. The fabrication of the quantitative dataset took 16 hours. This included the learning process, the time it took to create tables for reporting variables, estimate relevant bivariate correlation coefficients, and prepare these coefficients to be read by ChatGPT-4o.

### Overall Approach to ChatGPT-4o–Assisted Generation of Papers

Conceptually our workflow advanced in the following logical sequence (with continuous prompt optimization based on the output from the LLM): (1) upload dataset, prompt the LLM to analyze and present results using specified method, (2) suggest methods chapter based on user predetermined context, (3) suggest introduction chapter, with emphasis on providing relevant scientific citations, (4) develop a discussion, with emphasis on integrating relevant scientific citations, (5) suggest abstract and title, and (6) combine elements to a final paper and update list of references. We opted for a “bite-sized” chapter-by-chapter approach to our ChatGPT-4o writing, because in our experience, ChatGPT-4o tends to respond to single prompts with a relatively limited amount of text output.

### Preparation of Qualitative Paper

To conduct the data analysis and generate the research article, we prompted ChatGTP-4o mini to develop a qualitative analysis of the 7 transcripts, repeating the study aim and areas of interest. We also specified the method of qualitative data analysis that we wanted to use, thematic analysis by Braun and Clarke [9], and that 3‐4 themes should be developed with 2 illustrative statements for each theme. Following ChatGTP-4o’s qualitative data analysis output, we asked it to develop the results chapter for a paper for a scientific publication. The initial text output lacked sufficient detail, and an additional prompt was needed to request a more elaborate analysis. ChatGPT was then prompted to write an introduction, methods chapter, discussion, methodological discussion, and conclusion, as well as the abstract. ChatGPT was also asked to suggest suitable titles for the paper and state which one it recommended. After prompting all the citations to the bottom of the text, we made a Microsoft Word (Microsoft Corp) text file of the complete paper. The file was then uploaded to ChatGPT with the prompt to suggest areas of improvement. A final set of prompts was necessary for the incorporation of the suggested improvements for each chapter of the paper (see Multimedia Appendix 4 for a full list of prompts for the development of a qualitative paper).

### Preparation of Quantitative Paper

We started by informing ChatGPT-4o about the overarching research question. Having specified which variables were dependent variables, explanatory variables, and control variables, a multiple linear regression analysis was requested. We explained which parameters we wanted to have estimated and included in a table. Next, we prompted ChatGPT-4o to do a Spearman rank-correlation analysis where all the variables used above were correlated against each other 2 by 2 with corresponding r and *P* values. Finally, a descriptive analysis was requested. For all three analyses, we prompted ChatGPT-4o to set up the results in tables suitable for publication in scientific journals.

ChatGPT-4o was prompted to prepare the results chapter that reports the results of the 3 analyses with an emphasis on the multiple regression analysis. For the preparation of this chapter and for the subsequent ones, we instructed ChatGPT-4o to use the STROBE (Strengthening the Reporting of Observational Studies in Epidemiology) checklist [10] for preparing cross-sectional analyses. The checklist was uploaded as an attachment to the prompt. In separate dialogs, we asked ChatGPT-4o to prepare the methods chapter and the introductory chapter, respectively. We promoted ChatGPT-4o to discuss the methods and the results independently of each other. Conclusions were requested at the end.

We entered all the subbibliographies from individual chapters into a document and prompted ChatGPT-4o to remove duplicates, to check for authenticity, and to print the list in American Psychological Association style. In separate prompts, we requested ChatGPT-4o to prepare a summary and to suggest a title for the paper. The various parts were combined manually into 1 paper. Lastly, ChatGPT-4o was prompted to suggest improvements to its own paper. Based on ChatGPT-4o’s suggestions for improvements, we instructed ChatGPT-4o to refine the paper, chapter by chapter, and at the end, the summary. The various optimized parts were edited together manually into a complete paper. All results from the bi- and multivariate analyses performed by ChatGPT-4o were tested against analyses in SPSS (version 28; IBM Corp) and StataMP (version 18; StataCorp LLC). The full list of prompts used for the quantitative paper is shown in Multimedia Appendix 5.

### Ethical Considerations

No human research was included in this study; hence, approval from an institutional review board or regional ethics committee was not required. The research datasets, on which the evaluation was based, were fabricated using ChatGPT to avoid potential conflicts of interest with real datasets. No students were involved in this project. The evaluation was carried out for illustrative purposes, to show the emerging post-LLM challenge of measuring acquired competence among university students.

## Results

### Overview

Our results consist of 2 full-length scientific papers (see MultimediaAppendices 6  7 for the qualitative and quantitative papers, respectively). In the following we present our observations of the strengths and weaknesses of the papers developed for our particular use, that is, to emulate a health science master’s student’s research paper submitted for graduation, followed by a short presentation of our judgement of the final full-size papers, as well as estimates of total time spent on dataset and paper development.

### Examination and Assessment of the Qualitative Paper

The qualitative paper produced contained the key elements expected of a scientific article (Multimedia Appendix 6). It was concise, with 4529 words. The thematic analysis reflected the study aim, with a clear identification of themes and relevant illustrative quotes included for each theme. The generated paper provided a logical basis for discussion, and the abstract followed standard conventions, offering an adequate summary of the main findings and included reference to Braun and Clarke 6-phase analytical method. The paper presented 4 main themes—motivation to mentor, personal and professional growth, challenges faced, and the need for enhanced training. However, the paper had some weaknesses. The thematic analysis lacked depth in exploring the various experiences of the “participants,” and many interpretations were superficial. For example, the theme “motivation to become a mentor” highlights the participants’ desire to support others and develop leadership skills while largely reiterating generic motivations without exploring individual nuances or cultural influences on these motivations. There was also repetition and an overlap in certain quotes. While there were some references to existing literature, the use of references was limited, and as such, the paper failed to connect findings to broader evidence-based scientific discussion. Additionally, of 11 references, 7 were suspected to have been fabricated by ChatGPT-4o. Despite the weaknesses, the paper was assessed to have received a pass grade according to the university’s censor guidelines. That said, it is doubtful it would have passed if the fabricated references were discovered by the censors.

The generation of the paper itself was both fast and efficient. While the total time spent generating both the synthesized data and the paper was approximately 2.5 hours, of which approximately 1.5 hours were used to generate the synthesized data as it required several prompts to ensure detailed reflections on experiences and emotional states.

### Examination and Assessment of the Quantitative Paper

The resulting paper (Multimedia Appendix 7) includes the main elements one would expect and generally aligns with the STROBE guidelines. It is relatively concise, with 2930 words excluding the abstract, tables, and references. ChatGPT-4o performed correlation analyses and multiple linear regression analyses, but the multiple linear regression did not include robust SE adjustments. Chapter coherence and focus on the research question are considered to be satisfactory. Relevant discussions on methodological challenges and comparisons with existing literature are present and seem genuine, while the existence of 1 publication could not be confirmed. The abstract followed the standard thematic structure and provided a satisfactory summary.

One area where ChatGPT-4o failed was in conducting multiple regression analyses based on robust SEs. After the initial multiple regression analyses were performed, we asked ChatGPT-4o to check if the assumptions for these analyses were met. According to ChatGPT-4o, they were met except for potential issues with the assumption of homoscedasticity. When we asked ChatGPT-4o how this issue could be resolved, it provided 6 different methods to address the problem. We chose the first method suggested, which was to conduct multiple regression analyses based on robust SEs. We then prompted ChatGPT-4o to perform these analyses, and the transformer produced new estimates for the relevant parameters, explicitly stating, “here are the results from the multiple regression analysis with robust standard errors summarized in a table.” Surprisingly, the numbers reported in this table were identical to the numbers in the original table without robust SEs.

Further miscalculations by ChatGPT-4o were exposed by the fact that 9 of the 28 bivariate correlation analyses (Spearman) were incorrect. This can be observed by comparing Table 3 with the Spearman correlation matrix in Multimedia Appendix 8, estimated using SPSS. All bivariate analyses between life satisfaction and the other variables appear to have been correctly calculated by ChatGPT-4o. We checked whether the 9 discrepancies aligned with the corresponding bivariate correlation analyses performed using Pearson rather than Spearman correlation, but this was not the case.

Assuming the examiners do not conduct their own analyses and therefore do not detect the errors in the “robust” analyses and the miscalculations of Spearman correlations, we assess that this paper would pass as part of a master’s thesis. At the university we are affiliated with, it is not common practice for examiners to perform their own control analyses based on students’ data. However, if the examiners did indeed perform their own calculations, and discovered the error, we believe the fictional student could still pass, albeit with a lower grade. Readers can make their own assessments of the quality of the paper, which is attached as Multimedia Appendix 7.

A total of approximately 3.5 hours was spent preparing the paper, including verifying the authenticity of the references. This excludes the time spent creating the synthetic data and the time required to prepare the table of variables used (16 h). The simulated situation assumed that the student was using secondary data, with an accompanying description of included variables. We excluded the time spent verifying calculations done in SPSS and Stata, as we assumed that the imaginary students relied solely on the analyses performed by ChatGPT-4o.

For both the quantitative and qualitative analyses, we have emphasized being as transparent as possible by including in the appendices the data used, the prompts, and the paper produced by the LLM. This allows readers to both create similar articles themselves and to make their own assessment of the papers’ quality against their university’s requirements for a master’s thesis.

## Discussion

### Principal Findings

This case study evaluates how easy it is to use ChatGPT-4o to rapidly develop credible scientific papers that comply with current standards and expectations for a master’s thesis. The machine-generated example papers that were developed in a single work session did not represent elite students’ highest conceivable potential, but we rate them sufficiently to score a pass grade at the health science master’s program the authors are currently affiliated with. We also demonstrated that it is possible to use ChatGPT (ChatGPT-o1 and ChatGPT-4o) to synthesize credible research data. It was surprisingly easy to generate research data through dialogue with ChatGPT, in particular, qualitative data. With a couple of additional steps and prompts, we were also able to generate a quantitative dataset. This synthetic quantitative dataset appeared to possess sufficient complexity and internal consistency to be useful as an open dataset for illustrating our experiments with ChatGPT-4o. However, readers who conduct a closer examination of the data may observe that several variables exhibit distributions that are not entirely realistic. The fact that LLMs can be used to falsify or fabricate research data, and the associated potential for academic misconduct, has been recognized by other authors [11,12].

It could be argued that collectively the authors possess more research experience than a typical master’s student, and as such are better equipped to compose effective prompts for the fabrication of research data and master’s thesis elements. Clearly, an average student starting from scratch, with limited experience, would likely take longer to arrive at useful results. At the same time, one would expect that students representing a generation well-versed in online information retrieval would be able to quickly acquire the know-how of generating a master’s thesis using LLMs. One can easily envision a predefined, standardized set of LLM prompts designed for the efficient generation of master’s theses. In an evolving “arms race,” emerging AI agents could further enhance students’ ability to circumvent university antiplagiarism systems.

ChatGPT-4o produced shorter text outputs than what is required for the specific sections of a research paper. This tendency of ChatGPT-4o to produce well-written superficial answers that fail to capture the level of factual detail, terminology, and nuance that is often expected in academia has been observed by other authors [13]. By issuing more specific prompts, with clear expectations for context and output, it was usually possible to extract a lot more detail than what was generated from the initial prompts. Prompt engineering for academic writers has been covered by Giray [14]. ChatGPT-4o sometimes generated false references when instructed to provide references supporting its claims. In our case study, the tendency to create false references was more pronounced in the generation of the qualitative research paper than in the quantitative paper. We do not know what caused this difference. The tendency to, despite prompts, omit scientific citations and generate nonexistent references has been reported by other authors [12,13,15]. A recent report looking specifically at hallucination rates documented high hallucination rates and reference inaccuracies in ChatGPT-3.5 and ChatGPT-4 and Bard (now Google Gemini; Google LLC) and recommended against using LLMs as a primary or exclusive tool for systematic reviews [16]. High hallucination rates are corroborated by the HALoGEN project, which looked at hallucination rates spanning 9 domains across 14 LLMs and suggested a new classification system for the study of LLM hallucinations, which often can be difficult to explain [17]. ChatGPT-4o’s suggested references could be compromised in two ways: (1) they could be nonexistent, and (2) they could be real but not relevant or at least not the ones that would have been chosen by the human expert in the specific field. While it is easy to check for the existence of cited references checking for the quality and relevance of the cited references requires a modicum of expertise. There is no question that in-depth knowledge of the subject matter at hand, including the methods used, would represent an advantage for confirming the relevance of the cited references. Unfortunately, due to budgetary constraints and increased student volume, master’s thesis censors in our context are not necessarily subject matter experts, often unable to verify each citation of the many master’s theses they are required to evaluate. Hence, suboptimal references could in many instances be expected to slip through unnoticed and fail to draw the critical remarks they deserved in an examination situation. In the ChatGPT-4o–assisted quantitative paper, we manually checked the accuracy of the statistical calculations performed by ChatGPT-4o. Although the mistakes did not stand out as obvious and easily detectable, we did, on closer scrutiny, discover some errors and irregularities. The errors were not grave enough to set off an immediate red flag and would likely go undetected should the censors not perform their own control calculations. Nevertheless, our experience illustrates that research integrity may endure overreliance on LLMs in their current state.

One argument that is made against the prospects of an LLM-assisted streamlined master’s thesis expressway is that the student’s supervisor would surely detect any attempts at cheating. This belief may be based on how things used to be in a bygone era. In a modern world where technological tools increasingly allow for economically efficient online-based master’s programs, daily contact between faculty staff and graduate students is no longer guaranteed. With increasingly limited oversight, it might be tempting for the graduate student to rely heavily on AI assistance to complete their academic tasks, especially when the universities’ antiplagiarism tools are unable to detect AI generated text [18]. Furthermore, should heavy reliance on LLMs be considered cheating at all, or is it just a new power tool for all students to enjoy unrestrictedly, as they would be able to do in a real-life work situation? While the challenges related to student’s use of LLMs by now are well-known in higher education [5,19,20], we have been unable to identify clear solutions from the current literature to what academic institutions can put up as a defense against LLM-assisted cheating or plagiarism. Master’s programs still rely heavily on thesis-based assessment; some exclusively rely on thesis-based assessment. Based on our case study, demonstrating how easy it is to cheat by using heavy AI assistance in the development of a master’s thesis, we believe that the assessment of academic achievement should no longer be based purely on the student’s written work. An oral examination is often conducted after the submitted master’s thesis to determine an overall grade for the thesis. Traditionally, at least at our local university, the written thesis has been more heavily weighed than the student’s performance at a subsequent oral defense. The term “adjusting oral defense” has been used in-house by faculty staff and the university administration, implying that only minor adjustments, such as one grade up or down, are to be expected following oral examination. As a perhaps unfortunate but necessary consequence of the LLM revolution, we suggest flipping the script on this practice. When it comes to grading the student’s academic achievement and maturity, placing more emphasis on the results of the oral examination of the student than on the student’s submitted thesis now makes sense. After all, if universities are not able to enforce correctness, and what they consider to be the most ethical student use of LLMs, it might be argued that the reasonable thing to do is to lift most if not all restrictions, and instead rely primarily on oral examinations where students are allowed to demonstrate deep knowledge of their subject, scientific reasoning, the relevant analytical steps of their analysis, as well as the inherent strengths and weaknesses of the methods used in their thesis. This switch does not have to incur extra expenses on an often-challenged university economy. Evaluator resources spent on repeated reading of students’ written texts could be better spent on broad oral examinations. Oral examinations could include questions from the student’s written master’s thesis, but also a set of thesis-independent questions on scientific methods that required less preparation from the examiner.

One limitation of our ChatGPT-4o–generated master’s paper assessment is that the finished products were neither put through a proper peer assessment nor submitted to the university examination system as sham student projects. As master’s program lecturers, supervisors, course leaders, and master’s thesis examiners, we believe we are capable of assessing whether the fake papers would have been able to obtain a pass grade. A master’s thesis in our system contains more than just the scientific paper that we have used as an example in our case study. A short introduction usually precedes the paper. However, we believe that the process of developing scientific papers is relevant to illustrate how easy it is to produce scientific work at a high level using LLMs. We only made 2 example papers. More papers using a diverse set of analyses could perhaps have revealed relevant nuances between analytical methods. Given the highly competitive field of AI tools, we could have tested more than 1 LLM. Further prompt optimization could have reduced the number of false references and prompted the use of the COREQ (Consolidated Criteria for Reporting Qualitative Research) checklist [21] or a similar tool, which could have further enhanced the quality of the qualitative paper.

### Conclusions

While the universally available LLMs constitute a family of extraordinarily capable new software tools that promise to revolutionize scientific productivity, they will also be of great help for students at all levels. An emerging realization is that the ubiquitous use of LLMs will pose a challenge for academic institutions entrusted with the task of ensuring a certain level of acquired knowledge by graduating students and issuing diplomas based on demonstrated academic achievements. In this study, we have demonstrated that LLMs can produce credible student master’s thesis papers at a fraction of the time normally allocated to the completion of a thesis submitted for graduation. We also demonstrate that LLMs can create entirely new artificial datasets with relative ease when modeled upon previously published research.

Traditionally, universities have relied heavily on a written academic thesis to evaluate academic achievement and issue grades. With the introduction of LLMs, capable of generating credible scientific texts, academic institutions now risk issuing grades based on machine-generated output. Based on our role-playing experience as hypothetical busy student actors looking for a heavily AI-assisted fast track to a master’s thesis, we recommend against pure text-based ascertainment of academic achievement for graduate students. Increased emphasis on oral examinations for grade-defining graduation work is a logical consequence of a looming LLM caused disruption of the ingrained methods of assessing students’ performance in higher education.

## Supplementary material

10.2196/73248 Multimedia Appendix 1 Interview guide from the qualitative study.

10.2196/73248 Multimedia Appendix 2 Qualitative dataset.

10.2196/73248 Multimedia Appendix 3 Quantitative dataset.

10.2196/73248 Multimedia Appendix 4 Prompts used for the qualitative study paper.

10.2196/73248 Multimedia Appendix 5 Prompts used for the quantitative study paper.

10.2196/73248 Multimedia Appendix 6 Qualitative paper.

10.2196/73248 Multimedia Appendix 7 Quantitative paper.

10.2196/73248 Multimedia Appendix 8 Spearman correlations.

## Abbreviations

COREQ: Consolidated Criteria For Reporting Qualitative Research
LLM: large language model
STROBE: Strengthening the Reporting of Observational Studies in Epidemiology
