# Supplementary material for: Evaluating a Large Language Model’s Ability to Synthesize a Health Science Master’s Thesis: Case Study
Source: JMIR Form Res. 2025 Jul 3;9:e73248. doi: 10.2196/73248 (PMC12244274; doi:10.2196/73248)
Supplement: Multimedia Appendix 1 [file formative-v9-e73248-s001.docx]

**Interview Guide**

**Introduction:**

- Welcome the participant and thank them for their time.
- Explain the purpose of the interview: to explore their experiences as a student-peer mentor in the nursing mentoring program.
- Assure them that their responses will be kept confidential and used solely for research purposes.
- Obtain consent to record the interview for accuracy.
- Remind them that they can skip any questions they are uncomfortable answering.

**Questions:**

1. **Background Information:**
   - Can you tell me a bit about yourself? (Age, year of study)
   - Are you a registered nurse or a full-time student?
   - How long have you been a mentor in the program?
2. **Motivation and Expectations:**
   - What motivated you to become a student-peer mentor?
   - What were your initial expectations before starting this role?
3. **Experiences as a Mentor:**
   - How would you describe your overall experience in the mentoring program?
   - Can you share some highlights or memorable moments?
   - What challenges have you faced, and how did you overcome them?
4. **Impact on Personal and Professional Development:**
   - In what ways has being a mentor influenced your personal growth?
   - How has this experience impacted your professional development and nursing practice?
5. **Relationship with Mentees:**
   - How would you describe your relationships with your mentees?
   - Can you provide examples of successful interactions or breakthroughs?
6. **Support and Resources:**
   - What types of support did you receive from the program coordinators or institution?
   - Were there any resources or training that you found particularly helpful or lacking?
7. **Suggestions for Improvement:**
   - Based on your experience, what improvements would you suggest for the mentoring program?
   - What advice would you give to future student-peer mentors?
8. **Closing:**
   - Is there anything else you'd like to add about your experience as a student-peer mentor?
   - Thank the participant again for their time and insights.
