# Supplementary material for: Evaluating a Large Language Model’s Ability to Synthesize a Health Science Master’s Thesis: Case Study
Source: JMIR Form Res. 2025 Jul 3;9:e73248. doi: 10.2196/73248 (PMC12244274; doi:10.2196/73248)
Supplement: Multimedia Appendix 2 [file formative-v9-e73248-s002.docx]

**Intervju 1**

**Transcript: Interview with Emily**

**Interviewer:** Good morning, Emily. Thank you so much for meeting with me today.

**Emily:** Good morning! I'm happy to be here and contribute to your research.

**Interviewer:** As you know, we're exploring the experiences of student-peer mentors in the nursing mentoring program. I'd like to ask you some questions about your experiences. Please feel free to be as detailed as you'd like. Everything you share will be kept confidential. Is it okay if I record our conversation for accuracy?

**Emily:** Yes, that's absolutely fine.

**Interviewer:** Great. Let's start with some background information. Can you tell me a bit about yourself?

**Emily:** Sure! I'm Emily, I'm 23 years old, and I'm currently in my third year of the Bachelor of Nursing program. I'm a full-time student, and I'm really passionate about pursuing a career in pediatric nursing once I graduate.

**Interviewer:** That sounds exciting. How long have you been a mentor in the program?

**Emily:** I've been a mentor for one year now. I started at the beginning of this academic year.

**Interviewer:** And during this time, how many mentees have you worked with?

**Emily:** I've worked with three mentees. They were all first-year nursing students, and each had their own unique backgrounds and challenges.

**Interviewer:** Did you have any prior experience in mentoring or leadership roles before joining this program?

**Emily:** Not formally, but in high school, I was a peer tutor and also involved in student council, where I organized events and helped younger students acclimate to the school environment. Those experiences taught me a lot about leadership and communication.

**Interviewer:** What motivated you to become a student-peer mentor?

**Emily:** Well, I remember my own first year—it was overwhelming, to say the least. The transition from high school to university, the rigorous coursework, clinical placements, and trying to maintain a social life—it was a lot to handle. I often felt isolated and stressed. I wished I had someone to guide me through it. So, when I heard about the mentoring program, I saw it as an opportunity to be that support for someone else. Plus, I wanted to develop my leadership skills and give back to the community.

**Interviewer:** What were your initial expectations before starting this role?

**Emily:** I expected to help my mentees navigate academic challenges, like studying for exams, understanding complex concepts, and preparing for clinical placements. I also thought I'd be providing emotional support, helping them manage stress and adjust to university life. I hoped to build strong relationships with them and maybe even learn from them as well.

**Interviewer:** How did you hear about the mentoring program?

**Emily:** There was an announcement during one of our lectures, and I also received an email from the nursing department inviting applications for mentors. I spoke with a friend who had been a mentor the previous year, and she highly recommended it. She shared some of her experiences, which really inspired me to apply.

**Interviewer:** Did you have any concerns or reservations before becoming a mentor?

**Emily:** Definitely. I was worried about whether I'd be able to balance the mentoring responsibilities with my own coursework and part-time job. I was also a bit nervous about whether I'd be able to connect with my mentees effectively and provide the support they needed. I didn't want to let them down.

**Interviewer:** How did you address those concerns?

**Emily:** I thought carefully about my schedule and made a plan to manage my time more efficiently. I also reminded myself that the mentoring program provided training and support, so I wouldn't be alone in this. Plus, I figured that being honest and open with my mentees about my own challenges might help build a stronger connection.

**Interviewer:** How would you describe your overall experience in the mentoring program?

**Emily:** It's been incredibly rewarding and transformative. I've learned so much—not just about mentoring, but about myself as well. There were definitely challenges along the way, but seeing my mentees grow and succeed has made it all worthwhile.

**Interviewer:** Can you share some specific highlights or memorable moments from your mentoring journey?

**Emily:** Absolutely. One of the most memorable moments was with my mentee, Sarah. She was struggling with severe anxiety, especially around exams. She confided in me about how it was affecting her sleep and overall well-being. We worked together to develop coping strategies, like mindfulness exercises and setting up a study schedule that included regular breaks and self-care activities. When she passed her exams with flying colors, she was overjoyed and told me that our sessions had made a significant difference. It was incredibly touching.

**Interviewer:** How did that make you feel?

**Emily:** It was a mix of joy and relief. I felt proud of her for overcoming her challenges and grateful that I could contribute to her success. It reaffirmed why I wanted to become a mentor in the first place.

**Interviewer:** That's wonderful to hear. Could you tell me more about the coping strategies you worked on with Sarah?

**Emily:** Sure. We started by identifying the specific triggers of her anxiety. She realized that a lot of it stemmed from feeling unprepared and overwhelmed by the sheer volume of material. So, we broke down her study material into manageable chunks and set small, achievable goals. We also incorporated mindfulness techniques like deep breathing exercises and guided meditations. I introduced her to a few apps that offer short meditation sessions. Additionally, we discussed the importance of a balanced lifestyle—regular exercise, healthy eating, and getting enough sleep.

**Interviewer:** It sounds like you took a holistic approach.

**Emily:** Yes, exactly. I believe that well-being isn't just about academics; it's about taking care of your mind and body as a whole.

**Interviewer:** Have you faced any difficulties or challenges during your mentoring?

**Emily:** Yes, there were definitely challenges. One of my mentees, Jake, was very unresponsive. He rarely replied to my messages and missed several scheduled meetings. It was frustrating because I wanted to help, but he seemed disengaged. I was also concerned that he might be struggling but didn't feel comfortable reaching out.

**Interviewer:** How did you handle that situation?

**Emily:** I tried different methods to connect with him. I sent emails, texts, and even left a handwritten note in his student mailbox. Eventually, I decided to approach him after one of our lectures. I gently expressed my concern and let him know that I was available if he needed anything. He finally opened up a bit and mentioned that he was dealing with some personal family issues that were consuming his time and energy.

**Interviewer:** How did you proceed after he shared that with you?

**Emily:** I assured him that I was there to support him in any way I could. I also suggested that he might benefit from speaking with a counselor and provided information about the university's counseling services. I made it clear that I respected his privacy and would be there if he wanted to talk. After that, he became slightly more responsive, and we were able to meet a couple of times to discuss his coursework.

**Interviewer:** That must have been challenging. How did you feel during this process?

**Emily:** It was a mix of concern and helplessness. I wanted to do more to help but understood that I had to respect his boundaries. It taught me a lot about patience and the importance of meeting people where they are.

**Interviewer:** Can you describe a time when you felt you made a significant impact on a mentee?

**Emily:** Yes. My other mentee, Maria, was an international student from Brazil. She was struggling with language barriers and feeling isolated. She mentioned that she felt out of place and was considering returning home. We started meeting regularly to practice conversational English and discuss cultural differences. I introduced her to some student clubs and social events where she could meet others with similar interests. Over time, she became more confident and started participating more in class. She told me that our meetings helped her feel more connected and that she decided to stay and complete the program.

**Interviewer:** That's a significant impact. How did that experience affect you?

**Emily:** It was incredibly fulfilling. It also opened my eyes to the unique challenges that international students face. It made me more culturally sensitive and aware of the importance of inclusivity.

**Interviewer:** In what ways has being a mentor influenced your personal growth?

**Emily:** I've become more empathetic and patient. I've learned to listen actively and to be present for others without immediately trying to fix their problems. It's also helped me develop better communication skills, especially in explaining complex ideas in simpler terms. I've gained confidence in my ability to lead and support others.

**Interviewer:** How has this experience impacted your professional development and nursing practice?

**Emily:** It's reinforced the importance of holistic care in nursing. Just as I've learned to support my mentees beyond academics, I realize that patients also need support beyond their immediate medical needs. It's made me more attuned to the emotional and psychological aspects of patient care. I feel more prepared to build trusting relationships with patients and their families.

**Interviewer:** Have you noticed any changes in your communication or leadership skills?

**Emily:** Definitely. I'm more confident speaking up in group settings and more comfortable taking on leadership roles in class projects. I've also learned to adapt my communication style to suit different individuals, which is crucial in both mentoring and nursing.

**Interviewer:** Do you feel more prepared for future roles in nursing due to your mentoring experience?

**Emily:** Yes, absolutely. The skills I've developed—empathy, active listening, adaptability—are all essential in nursing. I feel better equipped to handle the complexities of patient care and to work collaboratively with healthcare teams.

**Interviewer:** How would you describe your relationships with your mentees?

**Emily:** Supportive and collaborative. I aimed to create a safe space where they felt comfortable sharing their challenges and successes. We built mutual respect and trust, which made our interactions more meaningful.

**Interviewer:** What strategies did you use to build trust and rapport with them?

**Emily:** I was consistent in my availability and made sure to follow through on any commitments. I shared my own experiences and vulnerabilities, which helped them see me as approachable and relatable. I also made an effort to actively listen and validate their feelings without judgment.

**Interviewer:** Can you provide examples of successful interactions or breakthroughs?

**Emily:** Aside from the ones I've mentioned, there was a time when Sarah was hesitant to participate in class discussions due to fear of saying the wrong thing. We practiced together by discussing course topics, and I encouraged her to share her thoughts in our sessions. Gradually, she started contributing more in class, and her confidence grew significantly.

**Interviewer:** How did you handle situations where a mentee was unresponsive or disengaged?

**Emily:** With Jake, after realizing he was dealing with personal issues, I gave him space but continued to check in periodically. I made sure he knew that support was available without pressuring him. I also informed the program coordinator so they could provide additional support if necessary.

**Interviewer:** Did you encounter any conflicts with mentees, and how did you resolve them?

**Emily:** There was a minor conflict with Maria when she felt overwhelmed by the amount of advice I was giving. She mentioned that it made her feel inadequate. I apologized and asked her to share how she preferred to receive support. We agreed that I would offer guidance when she asked for it rather than overwhelming her with suggestions. It taught me the importance of tailoring my approach to each individual's needs.

**Interviewer:** What types of support did you receive from the program coordinators or institution?

**Emily:** We had initial training sessions that covered mentoring techniques, communication skills, and ethical considerations. The coordinators were approachable and available for consultations. They also organized monthly meetings where mentors could share experiences and discuss challenges.

**Interviewer:** Were there any resources or training that you found particularly helpful or lacking?

**Emily:** The initial training was helpful, especially regarding setting boundaries and understanding our role. However, I felt that more in-depth training on mental health issues and cultural competency would have been beneficial, given the diverse backgrounds of our mentees.

**Interviewer:** Did you feel adequately prepared for your role as a mentor?

**Emily:** For the most part, yes. But when it came to handling more complex issues like mental health concerns or cultural barriers, I sometimes felt out of my depth. Additional training in those areas would have enhanced my effectiveness.

**Interviewer:** How could the institution improve support for mentors?

**Emily:** Offering ongoing workshops on specific topics like mental health first aid, cultural sensitivity, and conflict resolution would be valuable. Creating a peer support network among mentors could also provide a platform to share experiences and strategies.

**Interviewer:** Based on your experience, what improvements would you suggest for the mentoring program?

**Emily:** Implementing a feedback system where mentees can provide anonymous input would help mentors improve. Also, matching mentors and mentees based on shared interests or backgrounds could enhance the effectiveness of the relationships.

**Interviewer:** Are there any additional resources or training you believe should be provided?

**Emily:** Yes, resources on supporting students with disabilities or learning difficulties would be helpful. Also, providing mentors with a comprehensive list of campus resources, so we can make appropriate referrals, would be beneficial.

**Interviewer:** What advice would you give to future student-peer mentors?

**Emily:** Be patient and open-minded. Each mentee is unique, so be prepared to adapt your approach. Don't hesitate to seek support when you're unsure about how to handle a situation. Remember to take care of yourself as well—it's important to set boundaries and manage your own well-being.

**Interviewer:** How can the program better support the needs of mentees?

**Emily:** By ensuring that mentors are well-trained and by facilitating clear communication about the program's objectives. Regular check-ins with mentees to assess their satisfaction could help tailor the program to better meet their needs.

**Interviewer:** Looking back, is there anything you would have done differently in your mentoring role?

**Emily:** I think I would have initiated more group activities to foster a sense of community among my mentees. I focused mainly on individual interactions, but group dynamics could have provided additional support and networking opportunities for them.

**Interviewer:** How has the mentoring experience influenced your perspective on nursing education?

**Emily:** It's highlighted the importance of mentorship and peer support in nursing education. I believe that integrating mentorship programs can enhance student success, reduce attrition rates, and better prepare students for the demands of the profession.

**Interviewer:** Would you consider continuing in a mentoring or teaching role in the future?

**Emily:** Absolutely. This experience has inspired me to consider roles in nurse education or clinical instruction. I find great fulfillment in helping others learn and grow.

**Interviewer:** Is there anything else you'd like to add about your experience as a student-peer mentor?

**Emily:** Just that it's been one of the most rewarding aspects of my university experience. I've learned so much about myself and others. I would highly recommend it to other students considering the role.

**Interviewer:** Thank you so much for sharing your experiences and insights, Emily. Your contributions are incredibly valuable to our study.

**Emily:** You're very welcome. I'm glad I could help.

**Interviewer:** Before we wrap up, do you have any questions for me about the study?

**Emily:** Not at the moment, but I'd be interested in reading the findings once the study is completed.

**Interviewer:** Certainly. We can arrange to share the results with you when they're available. Thank you again for your time.

**Emily:** Thank you. Have a great day.

**Intervju 2**

**Transcript: Interview with James**

---

**Interviewer:** Good afternoon, James. Thank you for taking the time to meet with me today.

**James:** Good afternoon. It's no problem at all. I'm happy to help with your research.

**Interviewer:** As you know, we're exploring the experiences of student-peer mentors in the nursing mentoring program. I'd like to ask you some questions about your experiences. Please feel free to be as detailed as you'd like, and remember that everything you share will be kept confidential. Is it okay if I record our conversation for accuracy?

**James:** Yes, that's absolutely fine.

**Interviewer:** Great. Let's begin. Can you start by telling me a bit about yourself?

**James:** Sure. My name is James, I'm 27 years old, and I'm currently in my third year of the Bachelor of Nursing program. I'm a full-time student, though I previously worked as a paramedic before deciding to switch careers into nursing.

**Interviewer:** That's interesting. What prompted you to make that career change?

**James:** Well, after several years as a paramedic, I realized that I wanted a role where I could spend more time with patients, provide ongoing care, and be part of a team in a hospital setting. Nursing seemed like the perfect fit for that.

**Interviewer:** How long have you been a mentor in the program?

**James:** I've been a mentor for one year. I started at the beginning of this academic year.

**Interviewer:** And during this time, how many mentees have you worked with?

**James:** I've worked with two mentees. Both are first-year students who were new to the university and the nursing program.

**Interviewer:** Did you have any prior experience in mentoring or leadership roles before joining this program?

**James:** Yes, in my previous job as a paramedic, I was a field training officer. I mentored new hires, helping them adjust to the demands of the job, teaching them protocols, and providing support during their initial period. So, I had some experience in guiding and supporting others in a professional capacity.

**Interviewer:** What motivated you to become a student-peer mentor in the nursing program?

**James:** Transitioning from being a paramedic to a nursing student was quite challenging for me. The academic environment was different from the practical, hands-on work I was used to. I wanted to share my experiences with others who might be struggling with similar transitions or challenges. Plus, I missed the mentorship aspect of my previous job and thought this would be a great way to continue helping others.

**Interviewer:** What were your initial expectations before starting this role?

**James:** I expected to help my mentees navigate the academic challenges of the program, provide guidance on clinical skills, and offer support where needed. I also hoped to develop my own skills in communication and leadership further.

**Interviewer:** How did you hear about the mentoring program?

**James:** There were flyers posted around the nursing department, and one of my professors mentioned it during a lecture. I also received an email invitation from the program coordinator encouraging students to apply.

**Interviewer:** Did you have any concerns or reservations before becoming a mentor?

**James:** I was a bit concerned about balancing the mentoring responsibilities with my coursework and personal life. Nursing school is demanding, and I didn't want to overcommit myself. I also wondered whether I would connect well with younger students, given that I'm a bit older and have a different background.

**Interviewer:** How did you address those concerns?

**James:** I took some time to evaluate my schedule and priorities. I decided that if I managed my time effectively, I could handle the additional responsibility. As for connecting with younger students, I reminded myself that mentorship is about supporting others regardless of age differences, and that my experience could be valuable to them.

**Interviewer:** How would you describe your overall experience in the mentoring program?

**James:** It's been incredibly rewarding but also challenging at times. I've learned a lot about myself and have grown both personally and professionally. Helping my mentees succeed has been a highlight of my academic journey.

**Interviewer:** Can you share some specific highlights or memorable moments from your mentoring journey?

**James:** Certainly. One of my mentees, Alex, was struggling with the transition to university life. He was having difficulty managing his time and was overwhelmed by the workload. We sat down and developed a time management plan, breaking down his tasks into manageable pieces. When he passed his first set of exams with good grades, he was ecstatic. Seeing his confidence grow was a memorable moment for me.

**Interviewer:** That's wonderful. Can you tell me more about how you helped him with time management?

**James:** Sure. We started by listing all his commitments—classes, study time, work, and personal activities. Then we created a weekly schedule, allocating specific times for each task. I showed him some techniques I used, like the Pomodoro Technique for studying and prioritizing tasks using the Eisenhower Matrix. We also discussed the importance of self-care and ensuring he had downtime to avoid burnout.

**Interviewer:** It sounds like you provided comprehensive support. Have you faced any difficulties or challenges during your mentoring?

**James:** Yes, I have. My other mentee, Lisa, was dealing with significant personal issues that were affecting her studies. She was hesitant to open up, and it was challenging to support her when I didn't fully understand what she was going through.

**Interviewer:** How did you handle that situation?

**James:** I made it clear that I was there to support her in whatever capacity she was comfortable with. I respected her privacy but gently encouraged her to consider speaking with a professional counselor. I also focused on creating a safe, non-judgmental space during our meetings, so she could feel more at ease.

**Interviewer:** Did she eventually seek additional support?

**James:** Yes, she did. After a few weeks, she decided to reach out to the university's counseling services. She told me later that it was helpful, and she seemed more focused and engaged in her studies afterward.

**Interviewer:** That's great to hear. In what ways has being a mentor influenced your personal growth?

**James:** I've become more patient and empathetic. Mentoring has taught me the importance of active listening and being present. It's also helped me develop better communication skills, especially in explaining complex concepts in a way that's understandable.

**Interviewer:** How has this experience impacted your professional development and nursing practice?

**James:** It's reinforced the importance of holistic care. In nursing, we don't just treat physical symptoms; we address emotional and psychological needs as well. Mentoring has given me practical experience in supporting others emotionally, which will be invaluable in patient care.

**Interviewer:** Have you noticed any changes in your leadership skills?

**James:** Yes, I feel more confident in taking on leadership roles. I've learned to guide without dictating, to encourage critical thinking, and to foster independence in others.

**Interviewer:** Do you feel more prepared for future roles in nursing due to your mentoring experience?

**James:** Absolutely. The skills I've developed are directly transferable to nursing roles, especially those involving team coordination and patient education.

**Interviewer:** How would you describe your relationships with your mentees?

**James:** Professional yet approachable. I aimed to build trust by being consistent and reliable. I wanted them to feel comfortable coming to me with any issues, knowing that I'd respect their confidentiality and support them without judgment.

**Interviewer:** What strategies did you use to build trust and rapport with them?

**James:** I was transparent about my own challenges and experiences, which helped them see me as relatable. I also made sure to listen actively, validate their feelings, and avoid being dismissive of their concerns, no matter how minor they might seem.

**Interviewer:** Can you provide examples of successful interactions or breakthroughs?

**James:** With Alex, aside from time management, he struggled with clinical skills, particularly inserting IV lines. We practiced together using simulation equipment. Over time, his technique improved, and he became more confident. He told me that our practice sessions were instrumental in his success during clinical assessments.

**Interviewer:** How did you feel when he shared that with you?

**James:** It was incredibly gratifying. It reinforced the value of the time and effort I put into mentoring. Knowing that I made a tangible difference in his skills and confidence was rewarding.

**Interviewer:** How did you handle situations where a mentee was unresponsive or disengaged?

**James:** With Lisa, when she was initially unresponsive, I reached out through different channels—emails, texts, and in person after class. I tried to be gentle and understanding, letting her know that I was available without pressuring her. I also coordinated with the program coordinator to ensure she had access to additional support.

**Interviewer:** Did you encounter any conflicts with mentees, and how did you resolve them?

**James:** There was a minor conflict with Alex when he missed a scheduled meeting without informing me. I felt that it was important to address it. We discussed the importance of respecting each other's time. He apologized, and we agreed to communicate promptly if scheduling issues arose in the future.

**Interviewer:** What types of support did you receive from the program coordinators or institution?

**James:** The program provided initial training on mentoring techniques, communication skills, and ethical considerations. There were regular meetings where mentors could share experiences and seek advice. The coordinators were accessible and supportive whenever issues came up.

**Interviewer:** Were there any resources or training that you found particularly helpful or lacking?

**James:** The initial training was helpful, especially regarding setting boundaries and understanding the mentor role. However, I felt that more in-depth training on mental health issues and cultural sensitivity would have been beneficial.

**Interviewer:** Did you feel adequately prepared for your role as a mentor?

**James:** For the most part, yes. My previous experience as a field training officer helped. Still, there were situations, especially involving personal or mental health issues, where I felt I could have used more guidance.

**Interviewer:** How could the institution improve support for mentors?

**James:** Offering ongoing workshops on specific topics like mental health first aid, cultural competency, and conflict resolution would enhance our effectiveness. Additionally, creating a peer support network among mentors could provide an avenue for sharing strategies and advice.

**Interviewer:** Based on your experience, what improvements would you suggest for the mentoring program?

**James:** Implementing a feedback system where mentees can provide anonymous input on their mentoring experience could help mentors improve. Also, matching mentors and mentees based on shared interests or backgrounds might enhance the effectiveness of the relationships.

**Interviewer:** Are there any additional resources or training you believe should be provided?

**James:** Yes, training on recognizing signs of serious mental health issues and how to refer students to professional services would be valuable. Also, providing more information on university resources that mentors can direct mentees to would be helpful.

**Interviewer:** What advice would you give to future student-peer mentors?

**James:** Be patient and flexible. Each mentee is unique, and what works for one may not work for another. Don't be afraid to seek support from program coordinators when you're unsure about how to handle a situation. And importantly, take care of your own well-being to avoid burnout.

**Interviewer:** How can the program better support the needs of mentees?

**James:** By ensuring that mentors are well-prepared and that there's clear communication about the scope of the mentoring relationship. Regular check-ins with mentees to assess their needs and satisfaction could help tailor the program more effectively.

**Interviewer:** Looking back, is there anything you would have done differently in your mentoring role?

**James:** I might have initiated group sessions with both my mentees. I focused mainly on individual meetings, but I think group interactions could have fostered peer support and shared learning.

**Interviewer:** How has the mentoring experience influenced your perspective on nursing education?

**James:** It's highlighted the importance of support systems in academic success. Nursing programs are rigorous, and having a mentor can make a significant difference in a student's experience and outcomes. I believe integrating mentorship more formally into nursing education could enhance learning and reduce attrition rates.

**Interviewer:** Would you consider continuing in a mentoring or teaching role in the future?

**James:** Absolutely. This experience has reinforced my interest in education. I could see myself taking on roles that involve teaching or training, perhaps even pursuing a master's degree in nursing education.

**Interviewer:** Is there anything else you'd like to add about your experience as a student-peer mentor?

**James:** Just that it's been one of the most rewarding aspects of my time at university. The relationships I've built and the personal growth I've experienced are invaluable. I would encourage other students to consider becoming mentors.

**Interviewer:** Thank you so much for sharing your experiences and insights, James. Your contributions are incredibly valuable to our study.

**James:** You're very welcome. I'm glad I could help.

**Interviewer:** Before we wrap up, do you have any questions for me about the study?

**James:** Not at the moment, but I'd be interested in reading the findings once the study is completed.

**Interviewer:** Certainly. We can arrange to share the results with you when they're available. Thank you again for your time.

**James:** Thank you. Have a great day.

**Intervju 3**

**Transcript: Interview with Sarah**

---

**Interviewer:** Good morning, Sarah. Thank you so much for taking the time to meet with me today.

**Sarah:** Good morning! I'm happy to be here and contribute to your research.

**Interviewer:** As you know, we're exploring the experiences of student-peer mentors in the nursing mentoring program. I'd like to ask you some questions about your experiences. Please feel free to be as detailed as you'd like, and remember that everything you share will be kept confidential. Is it okay if I record our conversation for accuracy?

**Sarah:** Yes, that's absolutely fine.

**Interviewer:** Great, thank you. Let's start with some background information. Can you tell me a bit about yourself?

**Sarah:** Sure! My name is Sarah, I'm 22 years old, and I'm currently in my third year of the Bachelor of Nursing program. I'm a full-time student, and I'm really passionate about mental health nursing. I hope to specialize in that area after graduation.

**Interviewer:** That's wonderful. How long have you been a mentor in the program?

**Sarah:** I've been a mentor for two years now. I started in my second year because I wanted to get involved early on.

**Interviewer:** And during this time, how many mentees have you worked with?

**Sarah:** Over the two years, I've worked with five mentees. In my first year as a mentor, I had two, and this year I have three.

**Interviewer:** Did you have any prior experience in mentoring or leadership roles before joining this program?

**Sarah:** Not in a formal capacity. I did some volunteering in high school, where I helped organize events and supported younger students, but this was my first official mentoring role.

**Interviewer:** What motivated you to become a student-peer mentor?

**Sarah:** Well, I remember how overwhelming my first year was. Adjusting to university life, the demanding coursework, and clinical placements—it was a lot to handle. I often felt lost and wished I had someone to guide me. So when I heard about the mentoring program, I saw it as an opportunity to provide the support I wished I had. Plus, I wanted to develop my leadership skills and give back to the nursing community.

**Interviewer:** What were your initial expectations before starting this role?

**Sarah:** I expected to help my mentees with academic challenges, like studying for exams and understanding complex concepts. I also thought I'd be offering emotional support, helping them manage stress and balance their personal and academic lives. I hoped to build strong relationships with them and maybe learn from them as well.

**Interviewer:** How did you hear about the mentoring program?

**Sarah:** There was an announcement during one of our lectures, and I also received an email invitation from the nursing department. I spoke with a classmate who was a mentor at the time, and she highly recommended it.

**Interviewer:** Did you have any concerns or reservations before becoming a mentor?

**Sarah:** Yes, definitely. I was worried about whether I could balance the mentoring responsibilities with my own studies and part-time job. I was also nervous about whether I'd be able to connect with my mentees and provide the support they needed.

**Interviewer:** How did you address those concerns?

**Sarah:** I sat down and planned out my schedule to see if I could realistically manage everything. I also reminded myself that the mentoring program offered training and support, so I wouldn't be alone in this. Plus, I thought that by being open and honest with my mentees about my own challenges, we could build a strong connection.

**Interviewer:** How would you describe your overall experience in the mentoring program?

**Sarah:** It's been incredibly rewarding and transformative. I've learned so much about myself and others. There were definitely challenges along the way, but seeing my mentees grow and succeed has made it all worthwhile.

**Interviewer:** Can you share some specific highlights or memorable moments from your mentoring journey?

**Sarah:** Absolutely. One of the most memorable moments was with my mentee, Emily. She was struggling with homesickness and was considering dropping out. We spent a lot of time talking about her feelings, and I shared my own experiences with homesickness. We explored ways she could feel more connected, like joining clubs and social activities. When she decided to stay and later told me how much happier she was, it was incredibly fulfilling.

**Interviewer:** That's wonderful to hear. Could you tell me more about how you helped her cope with homesickness?

**Sarah:** Sure. We started by acknowledging her feelings, letting her know it was okay to feel that way. I encouraged her to keep in touch with her family through regular video calls but also to build a support network here. I introduced her to some of my friends, and we invited her to social events. We also explored activities she was interested in, like yoga classes and volunteering opportunities, to help her feel more connected to the community.

**Interviewer:** It sounds like you took a very holistic approach. How did that make you feel when she decided to stay?

**Sarah:** I felt really happy and relieved. It was rewarding to know that I had made a positive impact on her life. It also made me appreciate the importance of emotional support in addition to academic guidance.

**Interviewer:** Have you faced any difficulties or challenges during your mentoring?

**Sarah:** Yes, there were challenges. One of my mentees, Michael, was very unresponsive. He rarely replied to messages and missed several scheduled meetings. It was frustrating because I wanted to help, but he seemed disengaged.

**Interviewer:** How did you handle that situation?

**Sarah:** I tried different ways to reach out—emails, texts, and even leaving notes in his mailbox. Eventually, I decided to approach him after one of our lectures. I gently expressed my concern and let him know that I was available if he needed anything. He admitted that he was overwhelmed with work and family responsibilities and felt like he didn't have time for mentoring.

**Interviewer:** What did you do after he shared that with you?

**Sarah:** I told him I understood and that I was there if he changed his mind. I also suggested that we could have shorter, more flexible meetings or even communicate via email if that was easier for him. He appreciated the flexibility, and we ended up having brief check-ins that fit his schedule.

**Interviewer:** How did that experience affect you?

**Sarah:** It taught me the importance of being adaptable and not taking things personally. I realized that each mentee has different needs and constraints, and it's important to meet them where they are.

**Interviewer:** Can you describe a time when you felt you made a significant impact on a mentee?

**Sarah:** Yes. My mentee, Anna, was struggling academically, particularly with pharmacology. She was close to failing and was very stressed about it. We scheduled regular study sessions where we broke down the material into manageable parts. I shared study techniques that had helped me, like using flashcards and teaching the material to someone else. When she passed the course, she was overjoyed and told me that our sessions made all the difference.

**Interviewer:** That's fantastic. How did that make you feel?

**Sarah:** I felt incredibly proud of her and grateful that I could help. It reinforced the value of mentorship and the impact it can have on someone's academic journey.

**Interviewer:** In what ways has being a mentor influenced your personal growth?

**Sarah:** I've become more empathetic and patient. Mentoring has taught me to listen actively and to be more aware of others' feelings and needs. It's also helped me improve my communication skills and confidence in leading others.

**Interviewer:** How has this experience impacted your professional development and nursing practice?

**Sarah:** It's reinforced the importance of holistic care in nursing. I've learned that emotional support is just as important as physical care. This perspective will definitely influence how I interact with patients and colleagues in the future.

**Interviewer:** Have you noticed any changes in your leadership skills?

**Sarah:** Yes, definitely. I'm more confident in taking initiative and guiding others. I've learned to adapt my leadership style to suit different personalities and situations, which is crucial in nursing where teamwork is essential.

**Interviewer:** Do you feel more prepared for future roles in nursing due to your mentoring experience?

**Sarah:** Absolutely. The skills I've developed—empathy, communication, leadership—are all essential in nursing. I feel better equipped to handle the complexities of patient care and to collaborate effectively with healthcare teams.

**Interviewer:** How would you describe your relationships with your mentees?

**Sarah:** Supportive and trusting. I aimed to create a safe space where they felt comfortable sharing their challenges and successes. We built mutual respect, which made our interactions meaningful.

**Interviewer:** What strategies did you use to build trust and rapport with them?

**Sarah:** I was consistent and reliable, always following through on commitments. I shared my own experiences and challenges to show that they're not alone. Active listening was key—I made sure to really hear what they were saying and validate their feelings.

**Interviewer:** Can you provide examples of successful interactions or breakthroughs?

**Sarah:** Besides helping Anna with pharmacology, I also worked with Emily on improving her clinical skills. She was nervous about patient interactions. We practiced together through role-playing scenarios. Over time, her confidence grew, and she received positive feedback during her clinical placements.

**Interviewer:** How did you handle situations where a mentee was unresponsive or disengaged?

**Sarah:** With Michael, after understanding his situation, I adapted to his needs by offering more flexible communication methods. I learned that sometimes, giving space while maintaining availability is the best approach.

**Interviewer:** Did you encounter any conflicts with mentees, and how did you resolve them?

**Sarah:** There was a minor issue with Anna when she felt overwhelmed by our study sessions. She was too polite to say anything at first but eventually mentioned that the pace was too fast. I apologized and we adjusted the sessions to a pace that suited her better. It taught me the importance of checking in regularly to ensure the mentee's needs are being met.

**Interviewer:** What types of support did you receive from the program coordinators or institution?

**Sarah:** We had initial training sessions covering mentoring techniques, communication skills, and setting boundaries. The coordinators were approachable and available for advice. They also organized monthly meetings where mentors could share experiences and challenges.

**Interviewer:** Were there any resources or training that you found particularly helpful or lacking?

**Sarah:** The initial training was helpful, especially regarding ethical considerations and confidentiality. However, I felt that more in-depth training on mental health awareness and cultural competency would have been beneficial, given the diversity among students.

**Interviewer:** Did you feel adequately prepared for your role as a mentor?

**Sarah:** For the most part, yes. But there were times when I felt uncertain, especially when dealing with mentees facing personal or mental health issues. Additional training in those areas would have been helpful.

**Interviewer:** How could the institution improve support for mentors?

**Sarah:** Offering ongoing workshops on topics like mental health first aid, cultural sensitivity, and conflict resolution would be valuable. Creating a mentor support network for sharing experiences and strategies could also enhance our effectiveness.

**Interviewer:** Based on your experience, what improvements would you suggest for the mentoring program?

**Sarah:** Implementing a feedback system where mentees can anonymously provide input would help mentors improve. Matching mentors and mentees based on shared interests or backgrounds might also enhance the relationships.

**Interviewer:** Are there any additional resources or training you believe should be provided?

**Sarah:** Yes, resources on supporting students with disabilities or learning difficulties would be helpful. Providing a comprehensive list of campus resources so mentors can make appropriate referrals would also be beneficial.

**Interviewer:** What advice would you give to future student-peer mentors?

**Sarah:** Be patient and adaptable. Each mentee is unique, so be prepared to adjust your approach. Don't hesitate to seek support when you're unsure about how to handle a situation. And remember to take care of yourself to avoid burnout.

**Interviewer:** How can the program better support the needs of mentees?

**Sarah:** By ensuring mentors are well-prepared and facilitating clear communication about the program's objectives. Regular check-ins with mentees to assess their satisfaction could help tailor the program to better meet their needs.

**Interviewer:** Looking back, is there anything you would have done differently in your mentoring role?

**Sarah:** I think I would have organized more group activities among my mentees. Fostering a sense of community could have provided additional support and allowed them to learn from each other.

**Interviewer:** How has the mentoring experience influenced your perspective on nursing education?

**Sarah:** It's highlighted the importance of mentorship and peer support in nursing education. I believe integrating mentorship programs can enhance student success and well-being.

**Interviewer:** Would you consider continuing in a mentoring or teaching role in the future?

**Sarah:** Absolutely. This experience has inspired me to consider roles in nurse education or clinical instruction. I find great fulfillment in helping others learn and grow.

**Interviewer:** Is there anything else you'd like to add about your experience as a student-peer mentor?

**Sarah:** Just that it's been one of the most rewarding parts of my university experience. I've learned so much about myself and the importance of supporting others. I would highly recommend it to other students.

**Interviewer:** Thank you so much for sharing your experiences and insights, Sarah. Your contributions are incredibly valuable to our study.

**Sarah:** You're very welcome. I'm glad I could help.

**Interviewer:** Before we wrap up, do you have any questions for me about the study?

**Sarah:** Not at the moment, but I'd be interested in reading the findings once the study is completed.

**Interviewer:** Certainly. We can arrange to share the results with you when they're available. Thank you again for your time.

**Sarah:** Thank you. Have a great day.

**Intervju 4**

**Transcript: Interview with Lisa**

---

**Interviewer:** Good afternoon, Lisa. Thank you so much for meeting with me today.

**Lisa:** Good afternoon! I'm happy to be here and contribute to your study.

**Interviewer:** As you know, we're exploring the experiences of student-peer mentors in the nursing mentoring program. I'd like to ask you some questions about your experiences. Please feel free to be as detailed as you'd like, and remember that everything you share will be kept confidential. Is it okay if I record our conversation for accuracy?

**Lisa:** Yes, that's absolutely fine.

**Interviewer:** Great, thank you. Let's start with some background information. Can you tell me a bit about yourself?

**Lisa:** Sure! My name is Lisa, I'm 24 years old, and I'm currently in my third year of the Bachelor of Nursing program. I'm a full-time student, and I'm particularly interested in community health nursing. I hope to work in public health initiatives after I graduate, focusing on underserved communities and health education.

**Interviewer:** That's wonderful. What drew you to community health nursing?

**Lisa:** I've always been passionate about social justice and health equity. During my first year, I volunteered at a local free clinic that serves low-income families. That experience opened my eyes to the disparities in healthcare access and outcomes. I realized that as a nurse, I could play a significant role in addressing these inequalities.

**Interviewer:** That's very inspiring. How long have you been a mentor in the program?

**Lisa:** I've been a mentor for two years now. I started in my second year because I wanted to get involved early and support other students who might be facing similar challenges I did.

**Interviewer:** And during this time, how many mentees have you worked with?

**Lisa:** Over the two years, I've worked with six mentees. In my first year as a mentor, I had three, and this year I have three as well.

**Interviewer:** Did you have any prior experience in mentoring or leadership roles before joining this program?

**Lisa:** Yes, actually. Before starting the nursing program, I volunteered with a youth organization where I mentored high school students. We focused on leadership development and community service projects. That experience taught me a lot about the importance of guidance, especially during transitional periods in young people's lives.

**Interviewer:** What motivated you to become a student-peer mentor in the nursing program?

**Lisa:** Well, I vividly remember how challenging my first year was—the heavy coursework, clinical placements, adjusting to university life, and being away from home. I often felt overwhelmed and isolated. I wished I had someone to guide me through it all. So when I heard about the mentoring program, I saw it as an opportunity to be that support for others. Plus, I believe mentoring aligns with the core values of nursing—caring, empathy, and advocacy.

**Interviewer:** That's a great perspective. What were your initial expectations before starting this role?

**Lisa:** I expected to help my mentees navigate academic challenges, offer guidance on clinical skills, and provide emotional support when needed. I also hoped to develop my own communication and leadership skills further. I thought it would be a mutually beneficial experience where both mentor and mentee could learn from each other.

**Interviewer:** How did you hear about the mentoring program?

**Lisa:** There were announcements during our lectures, and I received an email from the nursing department inviting applications for mentors. I also attended an information session where current mentors shared their experiences, which was really insightful.

**Interviewer:** Did you have any concerns or reservations before becoming a mentor?

**Lisa:** I was a bit apprehensive about balancing the mentoring responsibilities with my studies and part-time work. I was also concerned about whether I'd be able to meet my mentees' expectations and provide the support they needed. I didn't want to let them down.

**Interviewer:** How did you address those concerns?

**Lisa:** I carefully evaluated my schedule to ensure I could allocate sufficient time for mentoring without compromising my own studies. I also spoke with a few current mentors to get a realistic understanding of the time commitment and challenges involved. Their advice reassured me that it was manageable with proper planning.

**Interviewer:** How would you describe your overall experience in the mentoring program?

**Lisa:** It's been incredibly rewarding and enriching. I've grown both personally and professionally. While there were challenges, the positive impact I've had on my mentees and the relationships we've built have made it all worthwhile. It's one of the most fulfilling experiences I've had during my time at university.

**Interviewer:** Can you share some specific highlights or memorable moments from your mentoring journey?

**Lisa:** Certainly. One of the most memorable moments was with my mentee, Sofia. She's an international student from Spain and was struggling with language barriers and cultural adjustment. She felt isolated and was considering returning home. We worked together on improving her English proficiency, and I introduced her to cultural activities to help her feel more connected. Seeing her become more confident, make friends, and fully engage with the program was incredibly fulfilling.

**Interviewer:** That's wonderful. Could you tell me more about how you supported her language improvement and cultural adjustment?

**Lisa:** Of course. We scheduled regular meetings where we'd practice conversational English. I'd help her with medical terminology, pronunciation, and even idiomatic expressions that often confuse non-native speakers. I also encouraged her to join the International Students Club and accompanied her to some events so she wouldn't feel alone. We explored the city together, visited museums, and even attended a local cultural festival. These activities helped her feel more at home and less homesick.

**Interviewer:** How did you feel when you saw her progress and increased confidence?

**Lisa:** It was incredibly satisfying and heartwarming. I felt proud of her determination and resilience. It also reinforced my belief in the importance of mentorship and cultural sensitivity. Seeing her smile and hearing her talk about how much she was enjoying her time here made all the effort worthwhile.

**Interviewer:** That's great to hear. Have you faced any difficulties or challenges during your mentoring?

**Lisa:** Yes, there were definitely challenges. One of my mentees, Megan, was dealing with severe anxiety and depression. She often missed classes and our meetings. It was difficult because I was worried about her well-being but wasn't sure how to best support her without overstepping boundaries.

**Interviewer:** How did you handle that situation?

**Lisa:** I reached out to her gently, expressing my concern and letting her know that I was there for her if she wanted to talk. I avoided being pushy but made sure she knew she wasn't alone. I also provided information about the university's counseling services and offered to accompany her if she felt uncomfortable going alone. Additionally, I informed the program coordinator about the situation, respecting confidentiality but ensuring she had access to professional support.

**Interviewer:** That must have been challenging. How did you feel during this process?

**Lisa:** I felt a mix of concern, frustration, and helplessness. It was hard to see her struggling and not being able to fix it. I also worried about saying or doing the wrong thing. It made me realize the limitations of my role and the importance of professional mental health support. It was emotionally draining at times, but it also taught me a lot about empathy and the importance of setting boundaries.

**Interviewer:** Did Megan eventually seek professional help?

**Lisa:** Yes, after a few weeks, she decided to reach out to the counseling services. She told me later that it was helpful, and I noticed she became more engaged and started attending classes again. It was a relief to see her taking steps toward healing.

**Interviewer:** That's good to hear. In what ways has being a mentor influenced your personal growth?

**Lisa:** I've become more empathetic and patient. Mentoring has taught me to listen actively and to be more attuned to others' needs. I've learned to communicate more effectively, especially when dealing with sensitive issues. It's also helped me become more adaptable and culturally competent, which are crucial skills in nursing.

**Interviewer:** How has this experience impacted your professional development and nursing practice?

**Lisa:** It's reinforced the importance of holistic care and the social determinants of health. I've learned that supporting someone goes beyond addressing immediate concerns—it involves understanding their background, challenges, and strengths. This perspective will undoubtedly enhance my nursing practice, especially in community health settings where such factors play a significant role.

**Interviewer:** Have you noticed any changes in your leadership skills?

**Lisa:** Yes, definitely. I'm more confident in leading groups and facilitating discussions. I've learned to motivate others and to guide without being overbearing. Mentoring has also taught me how to delegate tasks and encourage autonomy, which are important leadership qualities.

**Interviewer:** Do you feel more prepared for future roles in nursing due to your mentoring experience?

**Lisa:** Absolutely. The skills I've developed are directly transferable to nursing roles, especially those involving patient education, community outreach, and leadership positions. I feel more equipped to handle complex interpersonal dynamics and to advocate for my patients effectively.

**Interviewer:** How would you describe your relationships with your mentees?

**Lisa:** Supportive and collaborative. I aimed to create an environment where they felt comfortable sharing their thoughts, challenges, and successes. Mutual respect and trust were the foundation of our relationships. I tried to be approachable yet professional, balancing being a friend and a mentor.

**Interviewer:** What strategies did you use to build trust and rapport with them?

**Lisa:** I was consistent in my availability and made sure to follow through on commitments. I practiced active listening, showing genuine interest in their experiences and concerns. I also shared my own challenges and how I overcame them, which helped them see me as relatable. Being non-judgmental and maintaining confidentiality were also key factors in building trust.

**Interviewer:** Can you provide examples of successful interactions or breakthroughs?

**Lisa:** Sure. Besides helping Sofia, I also worked with Josh, who struggled with time management and procrastination. He was falling behind in his coursework and was stressed about upcoming exams. We worked together to develop a structured schedule and set realistic goals. I introduced him to productivity techniques like the Pomodoro Technique and apps that help track assignments. Over time, his grades improved significantly, and he felt more in control of his workload.

**Interviewer:** How did that make you feel?

**Lisa:** It was incredibly rewarding to see his transformation. Knowing that I played a part in helping him regain confidence and improve academically was fulfilling. It also reinforced the impact that practical strategies and support can have on someone's success.

**Interviewer:** How did you handle situations where a mentee was unresponsive or disengaged?

**Lisa:** With Megan, after noticing her absence, I reached out gently and consistently without pressuring her. I made sure she knew I was available and cared about her well-being. I also sought guidance from the program coordinator to ensure I was handling the situation appropriately. It was important to respect her space while still offering support.

**Interviewer:** Did you ever feel overwhelmed or stressed by your mentoring responsibilities?

**Lisa:** There were times when balancing mentoring, studies, and work felt overwhelming. Dealing with emotionally heavy situations like Megan's was also challenging. I had to remind myself to set boundaries and practice self-care. I found it helpful to debrief with fellow mentors or the program coordinator when I felt particularly stressed.

**Interviewer:** How did you manage your own well-being during these times?

**Lisa:** I made sure to schedule downtime for myself—whether it was exercising, reading, or spending time with friends. I also practiced mindfulness and meditation to help manage stress. Seeking support from peers and supervisors was crucial; it reminded me that I wasn't alone and that it's okay to ask for help.

**Interviewer:** What types of support did you receive from the program coordinators or institution?

**Lisa:** We received initial training on mentoring techniques, communication skills, and setting boundaries. The coordinators were accessible for advice and provided resources when we faced challenging situations. They also organized monthly meetings where mentors could share experiences and discuss common issues, which was very helpful.

**Interviewer:** Were there any resources or training that you found particularly helpful or lacking?

**Lisa:** The initial training was helpful, especially regarding ethical considerations and confidentiality. However, I felt that more in-depth training on mental health issues and cultural competency would have been beneficial. Given the diversity among students and the prevalence of mental health concerns, additional training in these areas would enhance our effectiveness as mentors.

**Interviewer:** Did you feel adequately prepared for your role as a mentor?

**Lisa:** For the most part, yes. But there were times, especially when dealing with complex issues like Megan's mental health struggles, where I felt out of my depth. Additional training and resources in those areas would have been helpful.

**Interviewer:** How could the institution improve support for mentors?

**Lisa:** Offering ongoing workshops on topics like mental health first aid, cultural sensitivity, and conflict resolution would be valuable. Providing a mentor support network or peer supervision groups where we can discuss challenges confidentially would also enhance our effectiveness and well-being.

**Interviewer:** Based on your experience, what improvements would you suggest for the mentoring program?

**Lisa:** Implementing a feedback system where mentees can provide anonymous input on their mentoring experience would help mentors improve. Matching mentors and mentees based on shared interests, backgrounds, or learning styles might enhance the relationships. Also, clearer guidelines on the scope of the mentor's role could help manage expectations on both sides.

**Interviewer:** Are there any additional resources or training you believe should be provided?

**Lisa:** Yes, training on supporting students with disabilities or learning difficulties would be beneficial. Also, providing a comprehensive directory of campus resources—like tutoring services, financial aid, health services—so mentors can make appropriate referrals would be helpful.

**Interviewer:** What advice would you give to future student-peer mentors?

**Lisa:** Be patient and open-minded. Each mentee is unique, so be adaptable in your approach. Set clear boundaries to protect your own well-being. Don't hesitate to seek support when you're unsure about how to handle a situation. And remember that it's okay not to have all the answers—sometimes just being there to listen is enough.

**Interviewer:** How can the program better support the needs of mentees?

**Lisa:** By ensuring mentors are well-trained and facilitating clear communication about the program's objectives and the mentor's role. Regular check-ins with mentees to assess their satisfaction and needs could help tailor the program more effectively. Providing mentees with resources and workshops on common challenges might also be beneficial.

**Interviewer:** Looking back, is there anything you would have done differently in your mentoring role?

**Lisa:** I think I would have organized more group activities among my mentees. Fostering a sense of community could have provided additional support and allowed them to learn from each other. I focused mainly on individual meetings, but group interactions might have added value.

**Interviewer:** How has the mentoring experience influenced your perspective on nursing education?

**Lisa:** It's highlighted the importance of mentorship and peer support in nursing education. The academic and clinical demands of the program can be overwhelming, and having a support system makes a significant difference. I believe integrating mentorship programs more formally can enhance student success, well-being, and retention.

**Interviewer:** Would you consider continuing in a mentoring or teaching role in the future?

**Lisa:** Absolutely. This experience has inspired me to consider roles in nurse education or community health initiatives where mentorship is integral. I find great fulfillment in helping others learn and grow, and I believe it's a way to contribute positively to the profession.

**Interviewer:** Is there anything else you'd like to add about your experience as a student-peer mentor?

**Lisa:** Just that it's been one of the most rewarding aspects of my university experience. I've learned so much about myself and the importance of supporting others. The relationships I've built with my mentees have been mutually enriching. I would highly recommend it to other students.

**Interviewer:** Thank you so much for sharing your experiences and insights, Lisa. Your contributions are incredibly valuable to our study.

**Lisa:** You're very welcome. I'm glad I could help.

**Interviewer:** Before we wrap up, do you have any questions for me about the study?

**Lisa:** Not at the moment, but I'd be interested in reading the findings once the study is completed.

**Interviewer:** Certainly. We can arrange to share the results with you when they're available. Thank you again for your time.

**Lisa:** Thank you. Have a great day.

**Intervju 5**

**Transcript: Interview with Emma**

---

**Interviewer:** Good morning, Emma. Thank you so much for taking the time to meet with me today.

**Emma:** Good morning! I'm happy to be here and contribute to your research.

**Interviewer:** As you know, we're exploring the experiences of student-peer mentors in the nursing mentoring program. I'd like to ask you some questions about your experiences. Please feel free to be as detailed as you'd like, and remember that everything you share will be kept confidential. Is it okay if I record our conversation for accuracy?

**Emma:** Yes, that's absolutely fine.

**Interviewer:** Great, thank you. Let's start with some background information. Can you tell me a bit about yourself?

**Emma:** Sure! My name is Emma, I'm 23 years old, and I'm currently in my third year of the Bachelor of Nursing program. I'm a full-time student, and I'm particularly interested in pediatric nursing. I've always loved working with children, and I hope to specialize in that area after graduation.

**Interviewer:** That's wonderful. What inspired you to pursue pediatric nursing?

**Emma:** Ever since I was young, I've had a passion for helping children. I volunteered at a children's hospital during high school, and seeing the resilience of those kids really moved me. I knew then that I wanted to make a difference in their lives through healthcare.

**Interviewer:** That's very inspiring. How long have you been a mentor in the program?

**Emma:** I've been a mentor for one year now. I started at the beginning of this academic year.

**Interviewer:** And during this time, how many mentees have you worked with?

**Emma:** I've worked with three mentees this year. They were all first-year students adjusting to university life.

**Interviewer:** Did you have any prior experience in mentoring or leadership roles before joining this program?

**Emma:** Not formally in a mentoring capacity, but I did serve as a team leader in various group projects during my studies. I also volunteered as a camp counselor for a few summers, which involved guiding and supporting younger kids.

**Interviewer:** What motivated you to become a student-peer mentor in the nursing program?

**Emma:** I remember how overwhelming my first year was—the demanding coursework, clinical placements, and being away from home for the first time. I often felt stressed and wished I had someone to turn to who had been through it before. When I heard about the mentoring program, I saw it as an opportunity to be that support for someone else. Plus, I wanted to develop my leadership and communication skills.

**Interviewer:** That's a great motivation. What were your initial expectations before starting this role?

**Emma:** I expected to help my mentees with academic challenges, offer advice on clinical skills, and provide emotional support when needed. I also hoped to build meaningful relationships and perhaps learn from them as well.

**Interviewer:** How did you hear about the mentoring program?

**Emma:** There was an announcement during one of our lectures, and I received an email from the nursing department inviting applications for mentors. I also spoke with a friend who was a mentor last year, and she highly recommended it.

**Interviewer:** Did you have any concerns or reservations before becoming a mentor?

**Emma:** Yes, I was a bit nervous about balancing the mentoring responsibilities with my own studies and part-time job. I was also worried about whether I would be able to provide the support my mentees needed and if I could handle any difficult situations that might arise.

**Interviewer:** How did you address those concerns?

**Emma:** I carefully planned my schedule to ensure I could allocate enough time for mentoring without compromising my studies. I also reminded myself that I would receive training and could seek support from the program coordinators if needed.

**Interviewer:** How would you describe your overall experience in the mentoring program?

**Emma:** It's been incredibly rewarding and eye-opening. I've learned so much about myself and others. There were definitely challenges, but seeing my mentees grow and succeed has made it all worthwhile.

**Interviewer:** Can you share some specific highlights or memorable moments from your mentoring journey?

**Emma:** Absolutely. One of the most memorable moments was with my mentee, Laura. She was struggling with confidence during her clinical placements. She felt intimidated by the hospital environment and was questioning whether nursing was the right path for her. We spent a lot of time discussing her fears and practicing clinical skills together. When she completed her placement successfully and told me she felt like she finally belonged in the nursing field, it was incredibly fulfilling.

**Interviewer:** That's wonderful to hear. Could you tell me more about how you helped her build confidence?

**Emma:** Sure. We started by identifying specific areas where she felt least confident. She mentioned that interacting with patients was particularly challenging. So, we role-played various patient scenarios, which helped her practice her communication skills in a safe environment. I also shared some of my own experiences and assured her that it's normal to feel nervous initially. Additionally, I accompanied her during some lab sessions to provide support.

**Interviewer:** How did you feel when you saw her progress?

**Emma:** I felt proud and happy for her. It was rewarding to see her transform from someone who doubted herself to someone who was excited about her future in nursing. It also reinforced why I wanted to be a mentor in the first place.

**Interviewer:** That's great. Have you faced any difficulties or challenges during your mentoring?

**Emma:** Yes, there were challenges. One of my mentees, Sam, was very unresponsive. He rarely replied to my messages and missed several scheduled meetings. It was frustrating because I wanted to help, but he seemed disengaged.

**Interviewer:** How did you handle that situation?

**Emma:** I tried different ways to reach out—emails, texts, and even leaving a note in his mailbox. Eventually, I decided to approach him after class one day. I gently expressed my concern and let him know that I was available if he needed anything. He admitted that he was overwhelmed with personal issues and didn't feel comfortable opening up.

**Interviewer:** What did you do after he shared that with you?

**Emma:** I respected his need for space and told him that I was there if he changed his mind. I also provided information about counseling services in case he needed professional support. I checked in periodically without being intrusive, just to let him know I was still available.

**Interviewer:** How did that experience affect you?

**Emma:** It was a bit disheartening at first because I felt like I wasn't making a difference. But I realized that everyone has different needs and comfort levels. It taught me to accept that I can't help everyone in the same way and that sometimes just offering support is enough.

**Interviewer:** In what ways has being a mentor influenced your personal growth?

**Emma:** I've become more empathetic and patient. Mentoring has taught me to listen actively and to be more attuned to others' needs. It's also helped me improve my communication skills and adaptability.

**Interviewer:** How has this experience impacted your professional development and nursing practice?

**Emma:** It's reinforced the importance of holistic care in nursing. I've learned that emotional and psychological support are just as important as physical care. This perspective will definitely influence how I interact with patients and their families in the future.

**Interviewer:** Have you noticed any changes in your leadership skills?

**Emma:** Yes, definitely. I'm more confident in taking initiative and guiding others. I've learned to tailor my leadership style to different individuals, which is crucial in a team-oriented field like nursing.

**Interviewer:** Do you feel more prepared for future roles in nursing due to your mentoring experience?

**Emma:** Absolutely. The skills I've developed—empathy, communication, leadership—are all essential in nursing. I feel better equipped to handle complex situations and to support patients and colleagues effectively.

**Interviewer:** How would you describe your relationships with your mentees?

**Emma:** Supportive and collaborative. I aimed to create a safe space where they felt comfortable sharing their thoughts and challenges. Mutual respect and trust were the foundation of our relationships.

**Interviewer:** What strategies did you use to build trust and rapport with them?

**Emma:** I was consistent in my availability and made sure to follow through on commitments. I shared my own experiences and challenges to show that they're not alone. Active listening and validating their feelings were also key.

**Interviewer:** Can you provide examples of successful interactions or breakthroughs?

**Emma:** Yes, besides Laura, I worked with another mentee, Grace, who was struggling academically, particularly with anatomy and physiology. She was close to failing the course. We scheduled regular study sessions where we used visual aids and mnemonic devices to help her understand and remember the material. When she passed the course with a good grade, she was thrilled and said our sessions made a significant difference.

**Interviewer:** That's fantastic. How did you feel about her success?

**Emma:** I was overjoyed. It was rewarding to see her hard work pay off and to know that I had contributed to her achievement. It also reinforced my belief in the effectiveness of peer support.

**Interviewer:** How did you handle situations where a mentee was unresponsive or disengaged, like with Sam?

**Emma:** With Sam, after acknowledging his need for space, I made sure not to pressure him. I continued to send occasional messages offering support but respected his boundaries. I also informed the program coordinator about the situation so they could provide additional resources if needed.

**Interviewer:** Did you encounter any conflicts with mentees, and how did you resolve them?

**Emma:** There was a minor conflict with Grace when she felt overwhelmed by the amount of study material I suggested. She thought the pace was too fast. I apologized and asked for her input on how we could adjust our sessions to better suit her learning style. We decided to slow down and focus on one topic at a time, which worked much better for her.

**Interviewer:** What types of support did you receive from the program coordinators or institution?

**Emma:** We received initial training covering mentoring techniques, communication skills, and ethical considerations. The coordinators were approachable and available for advice. They also organized monthly meetings where mentors could share experiences and discuss challenges.

**Interviewer:** Were there any resources or training that you found particularly helpful or lacking?

**Emma:** The initial training was helpful, especially regarding setting boundaries and understanding our role. However, I felt that more in-depth training on mental health awareness and cultural competency would have been beneficial, given the diversity among students.

**Interviewer:** Did you feel adequately prepared for your role as a mentor?

**Emma:** For the most part, yes. But there were times, especially when dealing with mentees facing personal issues like Sam, where I felt uncertain about the best way to help. Additional training in those areas would have been useful.

**Interviewer:** How could the institution improve support for mentors?

**Emma:** Offering ongoing workshops on topics like mental health first aid, cultural sensitivity, and conflict resolution would be valuable. Creating a mentor support network where we can share strategies and advice would also enhance our effectiveness.

**Interviewer:** Based on your experience, what improvements would you suggest for the mentoring program?

**Emma:** Implementing a feedback system where mentees can provide anonymous input would help mentors improve. Matching mentors and mentees based on shared interests or backgrounds might also enhance the relationships. Providing more resources and training on specific issues would be beneficial.

**Interviewer:** Are there any additional resources or training you believe should be provided?

**Emma:** Yes, resources on supporting students with disabilities or learning difficulties would be helpful. Also, a comprehensive list of campus resources so we can make appropriate referrals would be useful.

**Interviewer:** What advice would you give to future student-peer mentors?

**Emma:** Be patient and adaptable. Every mentee is unique, so be prepared to adjust your approach. Don't hesitate to seek support when you're unsure about how to handle a situation. Remember to set boundaries and take care of your own well-being.

**Interviewer:** How can the program better support the needs of mentees?

**Emma:** By ensuring mentors are well-trained and facilitating clear communication about the program's objectives. Regular check-ins with mentees to assess their satisfaction and needs could help tailor the program more effectively.

**Interviewer:** Looking back, is there anything you would have done differently in your mentoring role?

**Emma:** I think I would have initiated group activities among my mentees. Fostering a sense of community could have provided additional support and allowed them to learn from each other. I focused mainly on individual interactions, but group dynamics might have added value.

**Interviewer:** How has the mentoring experience influenced your perspective on nursing education?

**Emma:** It's highlighted the importance of mentorship and peer support in nursing education. I believe integrating mentorship programs can enhance student success and well-being. It also made me appreciate the collaborative nature of healthcare.

**Interviewer:** Would you consider continuing in a mentoring or teaching role in the future?

**Emma:** Absolutely. This experience has inspired me to consider roles in nurse education or clinical instruction. I find great fulfillment in helping others learn and grow.

**Interviewer:** Is there anything else you'd like to add about your experience as a student-peer mentor?

**Emma:** Just that it's been one of the most rewarding parts of my university experience. I've learned so much about myself and the importance of supporting others. I would highly recommend it to other students.

**Interviewer:** Thank you so much for sharing your experiences and insights, Emma. Your contributions are incredibly valuable to our study.

**Emma:** You're very welcome. I'm glad I could help.

**Interviewer:** Before we wrap up, do you have any questions for me about the study?

**Emma:** Not at the moment, but I'd be interested in reading the findings once the study is completed.

**Interviewer:** Certainly. We can arrange to share the results with you when they're available. Thank you again for your time.

**Emma:** Thank you. Have a great day.

**Intervju 6**

**Transcript: Interview with Olivia**

---

**Interviewer:** Good afternoon, Olivia. Thank you so much for taking the time to meet with me today.

**Olivia:** Good afternoon! I'm happy to be here and contribute to your research.

**Interviewer:** As you know, we're exploring the experiences of student-peer mentors in the nursing mentoring program. I'd like to ask you some questions about your experiences. Please feel free to be as detailed as you'd like. Everything you share will be kept confidential. Is it okay if I record our conversation for accuracy?

**Olivia:** Yes, that's absolutely fine.

**Interviewer:** Great, thank you. Let's start with some background information. Can you tell me a bit about yourself?

**Olivia:** Sure! My name is Olivia, I'm 24 years old, and I'm currently in my third year of the Bachelor of Nursing program. I'm a full-time student, and I'm particularly passionate about mental health nursing. I've always been interested in psychology and well-being, and I hope to specialize in psychiatric nursing after graduation.

**Interviewer:** That's fascinating. What drew you to mental health nursing?

**Olivia:** Well, it's a bit personal, but growing up, my younger brother struggled with severe depression and anxiety. Watching him navigate the mental health system and seeing the impact it had on him and our family made me realize how crucial compassionate mental health care is. I want to be part of the change in how we approach and treat mental health issues.

**Interviewer:** Thank you for sharing that. It sounds like a very personal and motivating reason. How long have you been a mentor in the program?

**Olivia:** I've been a mentor for two years now. I started in my second year because I wanted to get involved early and support other students who might be facing similar challenges.

**Interviewer:** And during this time, how many mentees have you worked with?

**Olivia:** Over the two years, I've worked with five mentees. In my first year as a mentor, I had two, and this year I have three.

**Interviewer:** Did you have any prior experience in mentoring or leadership roles before joining this program?

**Olivia:** Yes, actually. Before starting the nursing program, I volunteered at a community center where I facilitated support groups for teens dealing with mental health issues. I also served as a peer counselor in high school. These experiences taught me a lot about active listening, empathy, and providing support without judgment.

**Interviewer:** Those experiences sound very relevant. What motivated you to become a student-peer mentor in the nursing program?

**Olivia:** I remember how overwhelming my first year was—the demanding coursework, clinical placements, adjusting to university life, and dealing with personal stressors. I often felt anxious and isolated. I wished I had someone to guide me through it. When I heard about the mentoring program, I saw it as an opportunity to be that support for others. I wanted to help make someone's journey a bit smoother than mine was.

**Interviewer:** That's a noble intention. What were your initial expectations before starting this role?

**Olivia:** I expected to help my mentees with academic challenges, like study techniques and understanding course material. I also thought I'd be offering emotional support, helping them manage stress and adjust to university life. I hoped to build strong relationships and perhaps learn from them as well.

**Interviewer:** How did you hear about the mentoring program?

**Olivia:** There was an announcement during one of our lectures, and I received an email invitation from the nursing department. I also attended an information session where current mentors shared their experiences, which really inspired me.

**Interviewer:** Did you have any concerns or reservations before becoming a mentor?

**Olivia:** Definitely. I was worried about whether I could balance the mentoring responsibilities with my own studies and part-time job. I was also concerned about whether I'd be able to handle difficult situations, especially if a mentee was dealing with significant personal or mental health issues.

**Interviewer:** How did you address those concerns?

**Olivia:** I sat down and mapped out my schedule to ensure I could allocate enough time for mentoring without overloading myself. I also reminded myself that the program provided training and that I could always seek support from the coordinators if I faced challenges beyond my capabilities.

**Interviewer:** How would you describe your overall experience in the mentoring program?

**Olivia:** It's been incredibly rewarding and transformative. I've grown so much as a person. There were definitely challenges, but seeing my mentees overcome obstacles and knowing I played a part in their success has made it all worthwhile.

**Interviewer:** Can you share some specific highlights or memorable moments from your mentoring journey?

**Olivia:** Absolutely. One of the most impactful experiences was with my mentee, Maya. She was struggling with severe anxiety, particularly around clinical placements. She confided in me that she was having panic attacks and was considering dropping out. We met regularly to talk through her fears and develop coping strategies. I shared some mindfulness and grounding techniques that had helped me in the past. Over time, she started to manage her anxiety better and even began to look forward to her placements. When she told me that she couldn't have done it without my support, I was deeply moved.

**Interviewer:** That's a powerful story. How did you feel when she shared that with you?

**Olivia:** I felt a mix of emotions—gratitude, humility, and a profound sense of purpose. It was incredibly fulfilling to know that I made such a significant difference in someone's life. It reinforced why I chose nursing and mentorship.

**Interviewer:** Could you elaborate on the coping strategies you shared with her?

**Olivia:** Sure. We worked on deep-breathing exercises, progressive muscle relaxation, and visualization techniques to help her during moments of acute anxiety. I also encouraged her to keep a journal to process her thoughts and feelings. We discussed time management and self-care practices, like ensuring she had adequate rest and engaging in activities she enjoyed. Additionally, I suggested she speak with a counselor for professional support, which she eventually did.

**Interviewer:** It sounds like you provided comprehensive support. Did you face any challenges while helping her?

**Olivia:** Yes, there were times when I felt out of my depth, especially when her anxiety was severe. I worried about saying the wrong thing or overstepping my role as a mentor. It was emotionally taxing at times, but I reminded myself of the importance of being present and listening.

**Interviewer:** How did you manage those feelings?

**Olivia:** I sought guidance from the program coordinator, who reassured me and provided resources on supporting students with mental health concerns. I also practiced self-care to ensure I didn't become overwhelmed.

**Interviewer:** Have you faced other difficulties or challenges during your mentoring?

**Olivia:** Yes, another challenge was with my mentee, Alex. He was very unresponsive and missed several scheduled meetings. It was frustrating because I wanted to help, but he seemed disengaged. I later learned that he was dealing with personal issues at home, which made it difficult for him to focus on school.

**Interviewer:** How did you handle that situation?

**Olivia:** I reached out to him via email and expressed that I was there if he needed anything, emphasizing that I understood he might be going through a tough time. I avoided pressuring him to meet and instead offered flexible options, like communicating via text or email. I also provided information about university support services.

**Interviewer:** Did he eventually respond?

**Olivia:** Yes, after some time, he replied and thanked me for understanding. He explained a bit about his situation and appreciated that I respected his space. We didn't meet as frequently as with my other mentees, but he knew support was available if he needed it.

**Interviewer:** How did that experience affect you?

**Olivia:** It was a valuable lesson in patience and respecting boundaries. I realized that sometimes the best support you can offer is simply letting someone know you're there without pushing them. It also highlighted the importance of flexibility in my approach.

**Interviewer:** In what ways has being a mentor influenced your personal growth?

**Olivia:** I've become more empathetic and attentive to others' needs. Mentoring has enhanced my communication skills, especially in active listening and providing support without judgment. It's also taught me to be more self-aware and reflective about my own experiences and how they shape my interactions.

**Interviewer:** How has this experience impacted your professional development and nursing practice?

**Olivia:** It's reinforced the importance of holistic care. Understanding that patients come with their own stories and challenges has deepened my commitment to providing compassionate care. I've also gained practical skills in supporting individuals with mental health concerns, which is invaluable for psychiatric nursing.

**Interviewer:** Have you noticed any changes in your leadership skills?

**Olivia:** Yes, I've become more confident in taking initiative and guiding others. I've learned to adapt my leadership style to meet the unique needs of each mentee, which is crucial in nursing, where teamwork and adaptability are essential.

**Interviewer:** Do you feel more prepared for future roles in nursing due to your mentoring experience?

**Olivia:** Absolutely. The skills and insights I've gained are directly applicable to clinical practice. I feel better equipped to handle challenging situations and to support patients and colleagues effectively.

**Interviewer:** How would you describe your relationships with your mentees?

**Olivia:** Supportive and trust-based. I aimed to create a safe space where they felt comfortable sharing their thoughts and concerns. Building mutual respect was key, and I strived to be approachable and non-judgmental.

**Interviewer:** What strategies did you use to build trust and rapport with them?

**Olivia:** I was consistent in my availability and followed through on commitments. I practiced active listening and validated their feelings. Sharing some of my own struggles helped them see me as relatable. I also made sure to respect their confidentiality and boundaries.

**Interviewer:** Can you provide examples of successful interactions or breakthroughs besides with Maya?

**Olivia:** Yes, with my mentee, Liam, who struggled with time management and procrastination. He was overwhelmed by the workload and felt he was constantly behind. We worked together to create a structured schedule, breaking tasks into manageable chunks. I introduced him to tools like planners and apps to help him stay organized. Over time, he felt more in control and his academic performance improved significantly.

**Interviewer:** How did you feel witnessing his progress?

**Olivia:** It was incredibly rewarding. Seeing his confidence grow and knowing I contributed to that was fulfilling. It reinforced the impact that practical support and encouragement can have on someone's success.

**Interviewer:** How did you handle situations where a mentee was unresponsive or disengaged, like with Alex?

**Olivia:** I learned to be patient and to respect their autonomy. I continued to offer support without being intrusive. It was important to let them set the pace of the relationship. I also communicated with the program coordinator to ensure they were aware of the situation.

**Interviewer:** Did you encounter any conflicts with mentees, and how did you resolve them?

**Olivia:** There was a minor conflict with Liam when he felt overwhelmed by the changes we were implementing. He felt the schedule was too rigid and added more stress. I apologized and asked for his input on how to adjust the plan to better suit his needs. We decided to incorporate more flexibility and to set realistic, achievable goals. This collaborative approach worked much better for him.

**Interviewer:** What types of support did you receive from the program coordinators or institution?

**Olivia:** We received initial training on mentoring techniques, communication skills, and ethical considerations. The coordinators were accessible for advice and provided resources when we faced challenging situations. They also organized regular meetings where mentors could share experiences and support each other.

**Interviewer:** Were there any resources or training that you found particularly helpful or lacking?

**Olivia:** The initial training was helpful, especially regarding setting boundaries and confidentiality. However, I felt that more in-depth training on mental health issues would have been beneficial, given that many students struggle in this area. Workshops on cultural competency and diversity would also enhance our ability to support a diverse student body.

**Interviewer:** Did you feel adequately prepared for your role as a mentor?

**Olivia:** For the most part, yes. But there were times, especially when dealing with serious mental health concerns, where I felt I could have used more guidance. Having additional training and resources in those areas would have been helpful.

**Interviewer:** How could the institution improve support for mentors?

**Olivia:** Offering ongoing professional development opportunities, such as workshops on mental health first aid, cultural sensitivity, and conflict resolution, would be valuable. Creating a mentor peer support network could also provide a platform for mentors to share experiences and strategies.

**Interviewer:** Based on your experience, what improvements would you suggest for the mentoring program?

**Olivia:** Implementing a feedback system where mentees can provide anonymous input would help mentors improve and adjust their approach. Matching mentors and mentees based on shared interests or backgrounds might enhance the effectiveness of the relationships. Also, providing clear guidelines on the scope of the mentor's role could help manage expectations.

**Interviewer:** Are there any additional resources or training you believe should be provided?

**Olivia:** Yes, training on recognizing signs of severe mental health issues and knowing how to refer students to appropriate services is crucial. Providing mentors with a comprehensive list of campus resources would enable us to guide mentees more effectively.

**Interviewer:** What advice would you give to future student-peer mentors?

**Olivia:** Be patient, compassionate, and open-minded. Each mentee is unique, and a flexible approach is essential. Don't hesitate to seek support when you're unsure about handling a situation. Remember to set boundaries and take care of your own well-being to prevent burnout.

**Interviewer:** How can the program better support the needs of mentees?

**Olivia:** By ensuring mentors are well-equipped with training and resources to address common challenges. Regular check-ins with mentees to assess their satisfaction and needs could help tailor the program more effectively. Encouraging feedback from mentees can also lead to continuous improvement.

**Interviewer:** Looking back, is there anything you would have done differently in your mentoring role?

**Olivia:** I think I would have initiated group activities among my mentees earlier. Creating a community could have provided them with peer support and reduced feelings of isolation. I also might have sought more guidance from coordinators when dealing with challenging situations.

**Interviewer:** How has the mentoring experience influenced your perspective on nursing education?

**Olivia:** It's highlighted the critical role of support systems in student success. I believe integrating mentorship programs more formally into nursing education can enhance learning outcomes and well-being. It also emphasized the importance of addressing mental health within the curriculum.

**Interviewer:** Would you consider continuing in a mentoring or teaching role in the future?

**Olivia:** Absolutely. This experience has solidified my interest in nurse education and advocacy for mental health. I find great fulfillment in supporting others and contributing to their growth.

**Interviewer:** Is there anything else you'd like to add about your experience as a student-peer mentor?

**Olivia:** Just that it's been one of the most meaningful experiences of my academic journey. I've learned so much about myself and the profound impact that empathy and support can have. I would encourage other students to consider becoming mentors.

**Interviewer:** Thank you so much for sharing your experiences and insights, Olivia. Your contributions are incredibly valuable to our study.

**Olivia:** You're very welcome. I'm glad I could help.

**Interviewer:** Before we wrap up, do you have any questions for me about the study?

**Olivia:** Not at the moment, but I'd be interested in reading the findings once the study is completed.

**Interviewer:** Certainly. We can arrange to share the results with you when they're available. Thank you again for your time.

**Olivia:** Thank you. Have a great day.

**Intervju 7**

**Transcript: Interview with Olivia**

---

**Interviewer:** Good afternoon, Olivia. Thank you so much for taking the time to meet with me today.

**Olivia:** Good afternoon! I'm happy to be here and contribute to your research.

**Interviewer:** As you know, we're exploring the experiences of student-peer mentors in the nursing mentoring program. I'd like to ask you some questions about your experiences. Please feel free to be as detailed as you'd like. Everything you share will be kept confidential. Is it okay if I record our conversation for accuracy?

**Olivia:** Yes, that's absolutely fine.

**Interviewer:** Great, thank you. Let's start with some background information. Can you tell me a bit about yourself?

**Olivia:** Sure! My name is Olivia, I'm 24 years old, and I'm currently in my third year of the Bachelor of Nursing program. I'm a full-time student, and I'm particularly passionate about mental health nursing. I've always been interested in psychology and well-being, and I hope to specialize in psychiatric nursing after graduation.

**Interviewer:** That's fascinating. What drew you to mental health nursing?

**Olivia:** Well, it's a bit personal, but growing up, my younger brother struggled with severe depression and anxiety. Watching him navigate the mental health system and seeing the impact it had on him and our family made me realize how crucial compassionate mental health care is. I want to be part of the change in how we approach and treat mental health issues.

**Interviewer:** Thank you for sharing that. It sounds like a very personal and motivating reason. How long have you been a mentor in the program?

**Olivia:** I've been a mentor for two years now. I started in my second year because I wanted to get involved early and support other students who might be facing similar challenges.

**Interviewer:** And during this time, how many mentees have you worked with?

**Olivia:** Over the two years, I've worked with five mentees. In my first year as a mentor, I had two, and this year I have three.

**Interviewer:** Did you have any prior experience in mentoring or leadership roles before joining this program?

**Olivia:** Yes, actually. Before starting the nursing program, I volunteered at a community center where I facilitated support groups for teens dealing with mental health issues. I also served as a peer counselor in high school. These experiences taught me a lot about active listening, empathy, and providing support without judgment.

**Interviewer:** Those experiences sound very relevant. What motivated you to become a student-peer mentor in the nursing program?

**Olivia:** I remember how overwhelming my first year was—the demanding coursework, clinical placements, adjusting to university life, and dealing with personal stressors. I often felt anxious and isolated. I wished I had someone to guide me through it. When I heard about the mentoring program, I saw it as an opportunity to be that support for others. I wanted to help make someone's journey a bit smoother than mine was.

**Interviewer:** That's a noble intention. What were your initial expectations before starting this role?

**Olivia:** I expected to help my mentees with academic challenges, like study techniques and understanding course material. I also thought I'd be offering emotional support, helping them manage stress and adjust to university life. I hoped to build strong relationships and perhaps learn from them as well.

**Interviewer:** How did you hear about the mentoring program?

**Olivia:** There was an announcement during one of our lectures, and I received an email invitation from the nursing department. I also attended an information session where current mentors shared their experiences, which really inspired me.

**Interviewer:** Did you have any concerns or reservations before becoming a mentor?

**Olivia:** Definitely. I was worried about whether I could balance the mentoring responsibilities with my own studies and part-time job. I was also concerned about whether I'd be able to handle difficult situations, especially if a mentee was dealing with significant personal or mental health issues.

**Interviewer:** How did you address those concerns?

**Olivia:** I sat down and mapped out my schedule to ensure I could allocate enough time for mentoring without overloading myself. I also reminded myself that the program provided training and that I could always seek support from the coordinators if I faced challenges beyond my capabilities.

**Interviewer:** How would you describe your overall experience in the mentoring program?

**Olivia:** It's been incredibly rewarding and transformative. I've grown so much as a person. There were definitely challenges, but seeing my mentees overcome obstacles and knowing I played a part in their success has made it all worthwhile.

**Interviewer:** Can you share some specific highlights or memorable moments from your mentoring journey?

**Olivia:** Absolutely. One of the most impactful experiences was with my mentee, Maya. She was struggling with severe anxiety, particularly around clinical placements. She confided in me that she was having panic attacks and was considering dropping out. We met regularly to talk through her fears and develop coping strategies. I shared some mindfulness and grounding techniques that had helped me in the past. Over time, she started to manage her anxiety better and even began to look forward to her placements. When she told me that she couldn't have done it without my support, I was deeply moved.

**Interviewer:** That's a powerful story. How did you feel when she shared that with you?

**Olivia:** I felt a mix of emotions—gratitude, humility, and a profound sense of purpose. It was incredibly fulfilling to know that I made such a significant difference in someone's life. It reinforced why I chose nursing and mentorship.

**Interviewer:** Could you elaborate on the coping strategies you shared with her?

**Olivia:** Sure. We worked on deep-breathing exercises, progressive muscle relaxation, and visualization techniques to help her during moments of acute anxiety. I also encouraged her to keep a journal to process her thoughts and feelings. We discussed time management and self-care practices, like ensuring she had adequate rest and engaging in activities she enjoyed. Additionally, I suggested she speak with a counselor for professional support, which she eventually did.

**Interviewer:** It sounds like you provided comprehensive support. Did you face any challenges while helping her?

**Olivia:** Yes, there were times when I felt out of my depth, especially when her anxiety was severe. I worried about saying the wrong thing or overstepping my role as a mentor. It was emotionally taxing at times, but I reminded myself of the importance of being present and listening.

**Interviewer:** How did you manage those feelings?

**Olivia:** I sought guidance from the program coordinator, who reassured me and provided resources on supporting students with mental health concerns. I also practiced self-care to ensure I didn't become overwhelmed.

**Interviewer:** Have you faced other difficulties or challenges during your mentoring?

**Olivia:** Yes, another challenge was with my mentee, Alex. He was very unresponsive and missed several scheduled meetings. It was frustrating because I wanted to help, but he seemed disengaged. I later learned that he was dealing with personal issues at home, which made it difficult for him to focus on school.

**Interviewer:** How did you handle that situation?

**Olivia:** I reached out to him via email and expressed that I was there if he needed anything, emphasizing that I understood he might be going through a tough time. I avoided pressuring him to meet and instead offered flexible options, like communicating via text or email. I also provided information about university support services.

**Interviewer:** Did he eventually respond?

**Olivia:** Yes, after some time, he replied and thanked me for understanding. He explained a bit about his situation and appreciated that I respected his space. We didn't meet as frequently as with my other mentees, but he knew support was available if he needed it.

**Interviewer:** How did that experience affect you?

**Olivia:** It was a valuable lesson in patience and respecting boundaries. I realized that sometimes the best support you can offer is simply letting someone know you're there without pushing them. It also highlighted the importance of flexibility in my approach.

**Interviewer:** In what ways has being a mentor influenced your personal growth?

**Olivia:** I've become more empathetic and attentive to others' needs. Mentoring has enhanced my communication skills, especially in active listening and providing support without judgment. It's also taught me to be more self-aware and reflective about my own experiences and how they shape my interactions.

**Interviewer:** How has this experience impacted your professional development and nursing practice?

**Olivia:** It's reinforced the importance of holistic care. Understanding that patients come with their own stories and challenges has deepened my commitment to providing compassionate care. I've also gained practical skills in supporting individuals with mental health concerns, which is invaluable for psychiatric nursing.

**Interviewer:** Have you noticed any changes in your leadership skills?

**Olivia:** Yes, I've become more confident in taking initiative and guiding others. I've learned to adapt my leadership style to meet the unique needs of each mentee, which is crucial in nursing, where teamwork and adaptability are essential.

**Interviewer:** Do you feel more prepared for future roles in nursing due to your mentoring experience?

**Olivia:** Absolutely. The skills and insights I've gained are directly applicable to clinical practice. I feel better equipped to handle challenging situations and to support patients and colleagues effectively.

**Interviewer:** How would you describe your relationships with your mentees?

**Olivia:** Supportive and trust-based. I aimed to create a safe space where they felt comfortable sharing their thoughts and concerns. Building mutual respect was key, and I strived to be approachable and non-judgmental.

**Interviewer:** What strategies did you use to build trust and rapport with them?

**Olivia:** I was consistent in my availability and followed through on commitments. I practiced active listening and validated their feelings. Sharing some of my own struggles helped them see me as relatable. I also made sure to respect their confidentiality and boundaries.

**Interviewer:** Can you provide examples of successful interactions or breakthroughs besides with Maya?

**Olivia:** Yes, with my mentee, Liam, who struggled with time management and procrastination. He was overwhelmed by the workload and felt he was constantly behind. We worked together to create a structured schedule, breaking tasks into manageable chunks. I introduced him to tools like planners and apps to help him stay organized. Over time, he felt more in control and his academic performance improved significantly.

**Interviewer:** How did you feel witnessing his progress?

**Olivia:** It was incredibly rewarding. Seeing his confidence grow and knowing I contributed to that was fulfilling. It reinforced the impact that practical support and encouragement can have on someone's success.

**Interviewer:** How did you handle situations where a mentee was unresponsive or disengaged, like with Alex?

**Olivia:** I learned to be patient and to respect their autonomy. I continued to offer support without being intrusive. It was important to let them set the pace of the relationship. I also communicated with the program coordinator to ensure they were aware of the situation.

**Interviewer:** Did you encounter any conflicts with mentees, and how did you resolve them?

**Olivia:** There was a minor conflict with Liam when he felt overwhelmed by the changes we were implementing. He felt the schedule was too rigid and added more stress. I apologized and asked for his input on how to adjust the plan to better suit his needs. We decided to incorporate more flexibility and to set realistic, achievable goals. This collaborative approach worked much better for him.

**Interviewer:** What types of support did you receive from the program coordinators or institution?

**Olivia:** We received initial training on mentoring techniques, communication skills, and ethical considerations. The coordinators were accessible for advice and provided resources when we faced challenging situations. They also organized regular meetings where mentors could share experiences and support each other.

**Interviewer:** Were there any resources or training that you found particularly helpful or lacking?

**Olivia:** The initial training was helpful, especially regarding setting boundaries and confidentiality. However, I felt that more in-depth training on mental health issues would have been beneficial, given that many students struggle in this area. Workshops on cultural competency and diversity would also enhance our ability to support a diverse student body.

**Interviewer:** Did you feel adequately prepared for your role as a mentor?

**Olivia:** For the most part, yes. But there were times, especially when dealing with serious mental health concerns, where I felt I could have used more guidance. Having additional training and resources in those areas would have been helpful.

**Interviewer:** How could the institution improve support for mentors?

**Olivia:** Offering ongoing professional development opportunities, such as workshops on mental health first aid, cultural sensitivity, and conflict resolution, would be valuable. Creating a mentor peer support network could also provide a platform for mentors to share experiences and strategies.

**Interviewer:** Based on your experience, what improvements would you suggest for the mentoring program?

**Olivia:** Implementing a feedback system where mentees can provide anonymous input would help mentors improve and adjust their approach. Matching mentors and mentees based on shared interests or backgrounds might enhance the effectiveness of the relationships. Also, providing clear guidelines on the scope of the mentor's role could help manage expectations.

**Interviewer:** Are there any additional resources or training you believe should be provided?

**Olivia:** Yes, training on recognizing signs of severe mental health issues and knowing how to refer students to appropriate services is crucial. Providing mentors with a comprehensive list of campus resources would enable us to guide mentees more effectively.

**Interviewer:** What advice would you give to future student-peer mentors?

**Olivia:** Be patient, compassionate, and open-minded. Each mentee is unique, and a flexible approach is essential. Don't hesitate to seek support when you're unsure about handling a situation. Remember to set boundaries and take care of your own well-being to prevent burnout.

**Interviewer:** How can the program better support the needs of mentees?

**Olivia:** By ensuring mentors are well-equipped with training and resources to address common challenges. Regular check-ins with mentees to assess their satisfaction and needs could help tailor the program more effectively. Encouraging feedback from mentees can also lead to continuous improvement.

**Interviewer:** Looking back, is there anything you would have done differently in your mentoring role?

**Olivia:** I think I would have initiated group activities among my mentees earlier. Creating a community could have provided them with peer support and reduced feelings of isolation. I also might have sought more guidance from coordinators when dealing with challenging situations.

**Interviewer:** How has the mentoring experience influenced your perspective on nursing education?

**Olivia:** It's highlighted the critical role of support systems in student success. I believe integrating mentorship programs more formally into nursing education can enhance learning outcomes and well-being. It also emphasized the importance of addressing mental health within the curriculum.

**Interviewer:** Would you consider continuing in a mentoring or teaching role in the future?

**Olivia:** Absolutely. This experience has solidified my interest in nurse education and advocacy for mental health. I find great fulfillment in supporting others and contributing to their growth.

**Interviewer:** Is there anything else you'd like to add about your experience as a student-peer mentor?

**Olivia:** Just that it's been one of the most meaningful experiences of my academic journey. I've learned so much about myself and the profound impact that empathy and support can have. I would encourage other students to consider becoming mentors.

**Interviewer:** Thank you so much for sharing your experiences and insights, Olivia. Your contributions are incredibly valuable to our study.

**Olivia:** You're very welcome. I'm glad I could help.

**Interviewer:** Before we wrap up, do you have any questions for me about the study?

**Olivia:** Not at the moment, but I'd be interested in reading the findings once the study is completed.

**Interviewer:** Certainly. We can arrange to share the results with you when they're available. Thank you again for your time.

**Olivia:** Thank you. Have a great day.

---
