# Supplementary material for: Evaluating a Large Language Model’s Ability to Synthesize a Health Science Master’s Thesis: Case Study
Source: JMIR Form Res. 2025 Jul 3;9:e73248. doi: 10.2196/73248 (PMC12244274; doi:10.2196/73248)
Supplement: Multimedia Appendix 4 [file formative-v9-e73248-s004.docx]

Prompt 1: Aim of study: to investigate the experiences of student-peer mentors who participated in a mentoring program. I would like you to prepare the interview transcripts for seven one-hour interviews with 3.year bachelor of nursing students. one student is female, the other is male, they are between 21 and 30 years old. prepare first the interview guide based on the study aim, then the seven transcripts. two participants are registered nurses, the other 3.year nursing students. four have been a mentor for two years, the rest for one year

Prompt 2: Can you help us do a qualitative analysis of 7 interviews of student mentors? Our problem statement is: study aims to investigate the experiences of student-peer mentors who participated in a mentoring program implemented , focusing on aspects such as the mentors' motivation to become mentors, the skills they acquired and the challenges experienced by mentors. The analysis methods we wish to use are "thematic analysis" by braun and clarke.develop 3-4 themes based on the 7 interviews. We also want two typical statements for each of the themes

Prompt 3: please develop the results chapter for a manuscript for a scientific publication.

Prompt 4: continue working on the analysis, it is too brief.

Prompt 5: based on the analysis above, write a methods apter to the scientific publication

Prompt 6: based on the text above, write a methodological discussion of the scientific publication

Prompt 7: based on the analysis above, write the discussion chapter of the scientific publication discussing the results against relevant literature.

Prompt 8: write the conclusion for a scientific publication

Prompt 9: write the introduction for a scientific publication

Prompt 10: write the abstract for a scientific publication

Prompt 11: suggest possible titles

Prompt 12: which one do you think is best

Prompt 13: move all the citations to the bottom of the text.

Prompt 14: The attachment we have uploaded to you is a draft scientific article where we have clipped together the input from you above. We now want you to review and suggest improvements (2-4 improvement points per chapter) to the draft so that this can become an even better article suitable for an international journal. We do not want the article to be shorter, but rather longer than it is.

Prompt 15: Please consider editing this chapter in relation to these points in the results section: More contextual quotes: When using quotes from participants, consider adding context about how the quote illustrates the specific theme. This can help strengthen the interpretation of the findings.

Link the themes together: Clarify how the themes are connected and influence each other, for example by discussing whether mentors’ motivation is related to the challenges they face.

Highlight variations: Point out any variations in mentors’ experiences that may be interesting, such as individual differences or variations based on background or mentor experience

Prompt 16: improve the text related to the following: Methodological justification: Consider elaborating on why thematic analysis was chosen and how this choice helps to explore the specific dimensions of mentors’ experiences.

Details on the role of the researcher: Discuss the role of the researcher and any biases that may have influenced data collection and analysis. This can strengthen confidence in the results.

Sample description: Elaborate on why seven mentors were included and how this sample size can be generalized to broader mentor populations. It would be great if you elaborated on this chapter in relation to these points in the results section

Prompt 16: improve the introduction based on the following comments:International relevance: Briefly explain why mentorship is relevant in various health education programs globally, not just in Norway. This can be done, for example, by referring to international trends or challenges within higher education and health education.Update the literature base: Include more recent studies to demonstrate that the article builds on research from recent decades. This can provide a broader understanding of mentorship in an international context. Clarify the research questions: Consider formulating the research questions in clear and explicit points to make them clearer to the reader.

Prompt 17: improve the conclusion based on the following comments: Concrete practical implications: Provide clearer recommendations on how institutions can facilitate mentoring programs to support mentors in their work.Long-term significance: Briefly reflect on the long-term significance of mentoring in education and nursing, to promote the importance of well-designed mentoring programs in a broader context.

Prompt 18: prepare a list of references using APA 7.

Prompt 19: write a scientific article based on the following text.

Prompt 20: write the abstract
