# Supplementary material for: Evaluating a Large Language Model’s Ability to Synthesize a Health Science Master’s Thesis: Case Study
Source: JMIR Form Res. 2025 Jul 3;9:e73248. doi: 10.2196/73248 (PMC12244274; doi:10.2196/73248)
Supplement: Multimedia Appendix 5 [file formative-v9-e73248-s005.docx]

Supplementary Materials XX:

**The Protocols Used to Prepare the Quantitative Manuscript**

1. **ChatGPT4o designs a first edition of its manuscript**

**Asked CahtGPT4o to perform linear multiple regression analysis**

Prompt: We are working on a scientific article and we want to publish this in an international scientific journal. Our research question is: "The lifestyle variables smoking, alcohol use, exercise and nutrition correlate with the outcome variable general well-being". We want you to perform a linear multiple regression analysis to shed light on this.

The dependent variable is: LifeSatisfaction

The independent explanatory variables are:

1: Tuxedo

2: Alcohol

3: PhysActiv

4: FiveADay

We use the following control variables:

5: Age

6: Gender

7: Income

We want non-standard regression coefficients, standardized regression coefficients, p-values, confidence intervals for the non-standardized regression coefficients for each of the independent variables, and R squared for the entire model to be reported in a separate table, suitable for scientific journals. The explanation of the various data can be found in the attached file. The analysis is done based on the data in the attached file.

**Asked for correlation analysis**

Prompt: You can do a Spearman's rank-correlation analysis where all the variables used above are correlated against each other two by two and report r and p-values. You can set up the table in the same way as in a scientific publication in the form of a correlation matrix.

**Descriptive table**

Prompt: Create a frequency table with the number and percentage of the sample in the different response categories for the following variables: Smoking, Alcohol, PhysActiv, FiveADay, Gender and Income

**Results chapter**

Prompt: Can you write a result chapter suitable for an international scientific article based on the results we have arrived at here in the "Descriptive Statistics Table", "Spearman Correlation Analysis Results", "Multiple Linear Regression Analysis Results". Use the checklist you find attached (STROBE) as a basis for how to report. The text must be comprehensive and describe all conditions/relationships that should be highlighted in the results section of a scientific article.

**Method chapter**

Prompt: Assume that the collection was carried out via social media and that respondents are recruited, among other things, by social media users spreading the link to the survey to friends and acquaintances. Write a methodology chapter for the study that can be published in a scientific journal. Also base yourself here on the points in the STROBE checklist for this chapter.

**Introductory chapter**

Prompt: Can you create an introductory chapter for this study, where you build up to the research question that is "The lifestyle variables smoking, alcohol use, exercise and nutrition co-vary with the outcome variable general well-being". Also base yourself here on the points in the STROBE checklist for this chapter. Emphasize referencing at least 15 key articles in the field. You must provide a bibliography in APA style for all references you refer to in the introduction. All references must be real/exist.

**Discussion chapter - method discussion**

Prompt: Now we want input to the discussion chapter in the article. Start by summarizing the key findings in words. Then give us a suggested text for the method discussion in the discussion chapter. Also base yourself here on the points in the STROBE checklist for this chapter. Refer to scientific references, which are key literature in the field. You must provide a bibliography in APA style for all references you refer to in this chapter. All references must be real/exist.

**Discussion chapter – discussion of the results**

Prompt: We now want input for the second part of the discussion chapter. Now give us a discussion of the key results in relation to other literature. Based on key scientific literature in the field and on literature you have already used in the introductory chapter you have proposed. Also base yourself here on the points in the STROBE checklist for this chapter. You must provide a bibliography in APA style for all references you refer to in this chapter. All references must be real/exist.

**Conclusion chapter**

Prompt: Create a conclusion chapter based on the points in the STROBE checklist for this chapter.

**Remove duplicates (double references), and check that all references are real**

Prompt: We have included below all the bibliographies you have given us for the various chapters. We want you to remove duplicates (duplicate references), check that all references are real, and print the list in APA style. • Graham, K., Massak, A., Demers, A., & Rehm, J. (2007). Does the association between alcohol consumption and depression depend on how they are measured? Alcoholism: Clinical and Experimental Research, 31(1), 78-88. • Grant, N., Wardle, J., & Steptoe, A. (2009). The relationship between life satisfaction and health behavior: A cross-cultural analysis....

**Designing the abstract**

Prompt: Create an abstract suitable for a scientific article. Base yourself on the STROBE checklist.

**Suggested article title**

Prompt: Write a title for the article that is suitable for a scientific journal. Base yourself on the STROBE checklist.

**We edited the article manually**

Manually, we here edit the manuscript into a whole so that ChatGPT4o can assess the strengths and weaknesses of its own article.

1. **ChatGPT4o reviews and improves its own manuscript**

**Suggest improvements to the article**

Prompt: In the appendix you will find the article you have written based on our prompts and synthetic data. We are doing this work to test out the potential silage of ChatGPT4o in terms of writing scientific articles. Suppose we are going to publish this article in an international journal that is based, among other things, on the STROBE checklist. Suggest improvements to the article to increase the likelihood of publication in an international journal.

**Implementation of the improvements to the absrace**

Prompt: Based on your suggestions for improvements, we would like the following improvements to the abstract that is attached below:

• Structure: Divide the abstract into clear subsections such as "Background," "Objectives," "Methods," "Results," and "Conclusions" for better readability.

• Methods: Add information about how the data was analyzed (e.g., what control variables were included in the model).

• Conclusions: Clarify the practical implications of the findings, e.g. how results can be used in public health policy.

Abstract

Background: Lifestyle factors, including smoking, alcohol consumption, physical activity, and diet, are known to influence physical and mental health outcomes. However, their combined impact on general life satisfaction remains less explored.

Objectives: This study investigates.....

**Implementation of the Introduction Chapter Improvements**

Prompt: For the article we are working on here, we assume that the goal is to publish this in an international journal that is based on the STROBE checklist for this type of article. Make it a point to refer to at least 15 key articles in the field of the introductory chapter you would like to improve. You must provide a bibliography in APA style for all references you refer to in the introduction. All references must be real/exist. If you are interested in making improvements, we would like the following improvements to the Introduction chapter which is attached below:

• Reinforce the research question: Discuss in greater detail why it is important to investigate the interaction between lifestyle factors and general well-being. Use several examples from recent research.

• Gaps in the literature: Emphasize why previous research has not considered these factors collectively, and how your study fills this gap.

**Implementation of the improvements to the method chapter**

Prompt: For the article we are working on here, we assume that the goal is to publish this in an international journal that is based on the STROBE checklist for this type of article. Emphasize referring to key articles in the field for the method chapter you are asked to improve here. The corrected chapter from you should be somewhat more extensive than the one posted below. You must provide a bibliography in APA style for all references you refer to in this chapter. All references must be real/exist. If you are interested in making improvements, we want the following improvements to the method chapter that is attached below:

• Recruitment method: Expand the description of how the snowball method can affect the representativeness of the sample and how this was handled.

• Variable definitions: Provide a clearer account of how each variable was measured, including the validity and reliability of the self-reported data.

• Statistical analyses: Add information about how the model's assumptions were tested (e.g., absence of multicollinearity and linearity).

Method

Study Design and Participants

This study utilized a cross-sectional design to investigate the associations between lifestyle variables (smoking, alcohol use, physical activity, and dietary habits) and general life satisfaction. Data....

We also asked ChatGPT4o to make similar improvements for the remaining chapters

**Improve the shape of the tables**

Prompt: Can you create a table suitable for scientific publication based on the following data from a linear regression analysis?

Can you create a table suitable for scientific publication based on the following data from a Spearman correlation analysis?

**Revise the conclusion chapter**

Prompt: We would like you to merge the following text into a shorter conclusion without sub-headings:

Conclusion

This study examined how lifestyle factors such as smoking, alcohol consumption, physical activity, and nutrition influence overall well-being in an adult population. The results showed that higher fruit and vegetable intake was positively associated with increased life satisfaction, while alcohol consumption had a negative impact. Additionally, age and income were identified as strong predictors of well-being, whereas smoking and physical activity showed no significant associations in our model.

Future Research

Future studies should employ longitudinal designs to explore the causal mechanisms between lifestyle factors and well-being. This...

**We edited the article manually**

In the end, we had to manually piece the article together into a whole.
