# Supplementary material for: Evaluating a Large Language Model’s Ability to Synthesize a Health Science Master’s Thesis: Case Study
Source: JMIR Form Res. 2025 Jul 3;9:e73248. doi: 10.2196/73248 (PMC12244274; doi:10.2196/73248)
Supplement: Multimedia Appendix 6 [file formative-v9-e73248-s006.docx]

**Supporting and Empowering: Nursing Student Mentors and Their Experiences with Motivation, Challenges, and Relationship Building**

**Abstract**

**Background**

Mentorship programs are increasingly recognized as valuable tools for supporting students' academic and personal development, particularly in demanding fields such as nursing education. These programs address the growing need for healthcare professionals capable of managing clinical and interpersonal challenges, fostering skills like leadership, communication, and empathy. Despite the benefits, mentorship can be demanding, as mentors often balance academic responsibilities with the emotional demands of guiding diverse mentees.

**Methods**

This qualitative study examines the experiences of student mentors in nursing, focusing on their motivation, skill development, challenges, and relationship building with mentees. Data were collected through semi-structured interviews with seven mentors and analyzed using thematic analysis.

**Results**

Mentors reported dual motivations: personal growth and a desire to support others. They highlighted skill development in empathy, communication, and emotional intelligence, alongside challenges in time management and engaging unresponsive mentees. Strategies such as sharing personal experiences and active listening were used to build trust.

**Conclusions**

The study enhances understanding of mentorship in nursing education and underscores the need for institutional support, including mental health and cultural competence training. Recommendations include creating supportive networks for mentors and emphasizing emotional intelligence in mentor training to prepare future healthcare professionals for their roles.

**Introduction**

Mentorship programs have increasingly been recognized as valuable initiatives to support students' academic and personal development, especially in demanding programs such as healthcare and nursing education (Terrion & Leonard, 2007; Crisp & Cruz, 2009). These programs have gained international relevance as many countries face a growing demand for healthcare professionals capable of managing clinical and interpersonal challenges. Mentorship programs help meet these needs by enabling students to develop leadership, communication, and empathy skills (Colvin & Ashman, 2010). In nursing education, mentorship has become particularly vital, as mentors play a key role in helping students develop essential skills for patient care and interdisciplinary teamwork.

Research indicates that mentorship not only supports new students but also allows mentors to practice skills and reflect on social and emotional challenges they are likely to encounter in their future careers (Rhodes & DuBois, 2008). The global focus on patient-centered care has further highlighted the relevance of mentorship, as it prepares students to handle patient relationships with empathy and professional communication (Harrison & Leddy, 2021).

Despite the many benefits of mentorship, several studies report that the mentor role can be demanding and challenging. Mentors often struggle to balance academic commitments with mentoring tasks and emotional demands (Allen et al., 2004; Terrion & Leonard, 2007). Many mentors encounter mentees with diverse needs, including psychosocial challenges and cultural differences, requiring high adaptability and emotional resilience (Crisp & Cruz, 2009). Recent studies also emphasize that mentors may experience exhaustion or emotional strain, suggesting a need for more structured support systems (Steele et al., 2022; Wang et al., 2020).

There is a clear need to deepen the understanding of how mentors in healthcare education globally experience and manage the mentoring role, as well as what factors influence their motivation, skill development, and challenges in working with mentees. Few studies have explored how mentorship impacts mentors' development and coping strategies, and there is a need to investigate how mentorship programs can be structured to provide mentors with the support they need to succeed.

This study addresses this research gap by examining the experiences of student mentors in a nursing mentorship program, with a particular focus on motivation, skill development, emotional challenges, and relationship building. The research is based on thematic analysis of semi-structured interviews, providing deeper insights into the mentoring role.

The study aims to answer the following research questions:

1. What motivates student mentors to take on the mentor role?
2. How do student mentors develop skills through the mentoring role?
3. What challenges do student mentors face in their relationships with mentees, and how do these affect their coping strategies?

The purpose of the study is to contribute to a deeper understanding of mentorship in higher education, focusing on how mentorship programs can be designed to support both mentors and mentees in demanding academic environments such as nursing education. The findings will be relevant for institutions aiming to develop sustainable mentorship programs that promote an inclusive and supportive learning culture for future healthcare professionals globally.

**Methodology**

**Study Design**

This study employs a qualitative research method to explore the experiences of student mentors in a mentorship program, focusing on their motivations, skills, challenges, and experiences in building relationships with mentees. A qualitative approach was chosen to enable an in-depth exploration of personal and subjective experiences, which was essential for understanding the complex emotional and social aspects of mentorship (Kvale & Brinkmann, 2015). Thematic analysis was selected as the analytical approach, allowing the researchers to capture the various dimensions of the mentoring role and how these vary across individuals. Thematic analysis is particularly suited for studies aiming to identify and highlight underlying patterns in qualitative data (Braun & Clarke, 2006), making it appropriate for this study's objective of uncovering diverse mentor experiences.

**Data Collection**

Data were collected through semi-structured, in-depth interviews with seven student mentors participating in the mentorship program for healthcare and nursing students. Semi-structured interviews were chosen to allow for spontaneous and detailed descriptions and reflections, while the interview guide ensured that relevant topics were explored systematically. The interviews covered key themes such as mentors' motivation, skill development, challenges in the mentor role, and their relationship with mentees. Interviews were conducted face-to-face and lasted between 45 and 60 minutes, providing the researcher with an opportunity to explore participants' thoughts and feelings in depth.

**Data Collection (continued)**

A sample size of seven mentors was deemed sufficient to ensure diversity in experiences while enabling a thorough analysis of each interview. This sample provided insights from mentors with varying backgrounds and experiences. Although generalization to all mentor populations is not possible, the sample offers adequate depth to identify key themes that may be relevant in broader contexts. A larger sample could have provided additional variability, but the study's qualitative design prioritizes depth over breadth, supporting the chosen sample size.

**Analysis Strategy**

The analysis was conducted using thematic analysis as described by Braun and Clarke (2006). Thematic analysis was selected for its flexibility and structure, enabling the researcher to identify and organize patterns (themes) in qualitative data systematically (Braun & Clarke, 2013). This methodological approach allowed the study to focus on both individual differences and common themes, making it particularly useful in exploring the diverse experiences of mentors.

Braun and Clarke's six steps for thematic analysis were followed:

1. **Familiarization:** Transcripts were read multiple times to ensure a thorough understanding of the content, with preliminary reflections noted.
2. **Generating Codes:** A systematic coding of each interview was conducted, identifying meaningful units in the text and labeling them with codes. The codes represented various aspects of mentors' experiences, such as motivation, challenges, and skill development.
3. **Searching for Themes:** Codes were analyzed for patterns, and similar codes were grouped into preliminary themes. This process involved careful review to ensure the themes accurately represented the data in a consistent and meaningful way.
4. **Reviewing Themes:** Themes were evaluated against the overall dataset to ensure they were representative of the interview content. This involved assessing whether the themes aligned with each code and whether they provided a cohesive understanding of mentors' experiences.
5. **Defining and Naming Themes:** Themes were refined and defined to capture the essence of mentors' experiences concisely and clearly.
6. **Writing Up:** The analysis was presented through a narrative discussion, with each theme supported by quotes from participants to exemplify their experiences.

Table illustrating the analytical process:

| **Stage of Analysis** | **Raw Data** | **Code** | **Preliminary Theme** |
| --- | --- | --- | --- |
| Excerpt from Interview | "I wanted to give back and support someone who might feel as uncertain as I did in the beginning." | Motivated by personal experiences as a new student | Motivation to become a mentor |
| Excerpt from Interview | "I truly developed my ability to actively listen and build trust with my mentees." | Skills in communication and trust-building | Acquired skills |
| Excerpt from Interview | "Sometimes it felt challenging to balance time between my studies and the mentor role." | Time management and balance | Challenges as a mentor |

**Researcher’s Role and Reflexivity**

The researcher played an active role in both data collection and analysis, necessitating an awareness of how personal assumptions and perspectives could influence interpretations. Reflexivity was emphasized throughout the research process, with the researcher maintaining a journal to reflect on assumptions and expectations, particularly during the coding and theme identification phases. Recognizing the researcher’s role strengthens the study's credibility by demonstrating an awareness of how subjectivity may influence findings. To ensure inter-rater reliability, an external colleague with experience in thematic analysis reviewed and validated the analysis, ensuring that the findings were grounded in the data.

**Ethical Considerations**

The study adhered to ethical guidelines for qualitative research (NESH, 2016). Participants were informed of the study's purpose and how the data would be handled, and written consent was obtained before the interviews. To safeguard participants' privacy, transcripts were anonymized during the analysis process, and audio recordings were securely stored and scheduled for deletion after the study's completion. Respect for participants' integrity and their right to withdraw from the study at any time was emphasized throughout the research process.

**Validity and Reliability**

To ensure the study's validity and reliability, the entire analysis was reviewed and discussed with a colleague experienced in thematic analysis. This collaboration ensured that interpretations were well-founded in the data and minimized the risk of researcher bias. Reliability was maintained through a systematic approach to coding and theme identification, as well as by using quotes to support the analysis.

**Results**

**Motivation to Become a Mentor**

Mentors’ motivations were multifaceted, strongly tied to a desire to help others and achieve personal growth. Many mentors expressed a genuine desire to contribute to a safer and more supportive learning environment, helping new students transition to university. Several mentors had personally experienced challenges during their own first year and wanted to give back. Emily shared how her first year was marked by academic pressure and loneliness, driving her to support others in similar situations:

*"I remember how difficult my first year was—the transition from high school, the intense academic pressure, and feeling alone. I wanted to be the person who could support someone through those same challenges."*

Similarly, Sarah emphasized the importance of being a support system for others, reflecting on her own experiences of wishing for guidance as a new student:

*"It’s important to me to be there for someone else, just as I wished I had someone to support me at the beginning."*

For many mentors, the role was also an opportunity to develop their own skills and achieve personal growth, particularly in leadership, communication, and empathy. Some highlighted this aspect as a way to prepare for future professional roles. James described his aspiration to enhance his leadership and communication abilities for his nursing career:

*"I had a strong desire to strengthen my leadership skills and improve my communication, especially with future nursing roles in mind."*

Lisa saw the mentoring role as a chance to work on personal weaknesses and build skills essential for her professional life:

*"The mentor role gives me the opportunity to work on my own weaknesses and develop skills that will be crucial in my career."*

This dual motivation—to support others while fostering personal growth—served as a driving force, encouraging mentors to invest time and energy in the role despite the significant challenges they faced.

**Skill Development and Personal Growth**

Through the mentor role, many mentors experienced significant development of personal and professional skills, particularly in empathy, communication, and emotional intelligence. The role seemed to provide them with a deeper understanding of others’ perspectives and challenges. Olivia described how mentorship taught her to listen without judgment and to be present without trying to fix everything:

*"Being a mentor has taught me to listen without judgment and to be present without trying to fix everything. It has made me more aware of what it means to support someone holistically."*

Emma noted that she had become more patient and empathetic, which she saw as invaluable for her future nursing role:

*"I’ve become more patient and empathetic. I’ve learned to adapt communication to each mentee, which is invaluable in nursing."*

Many mentors also reported that the role strengthened their ability to convey academic knowledge in an accessible way, a skill they found would be useful when working with patients. Sarah shared how she learned to explain complex topics simply, adapting to different levels of understanding:

*"I’ve learned to explain complex topics in simple terms and adapt to different levels of understanding. This will be essential when working with patients."*

Through mentorship, most mentors felt they developed skills that would be beneficial in both their studies and future professional roles.

**Challenges in the Mentoring Role**

Mentors encountered several challenges in their role. A common experience was the difficulty of balancing mentoring responsibilities with studies and other commitments, which sometimes led to exhaustion. Lisa described her concern about managing all demands and how she found the responsibility challenging:

*"I was worried about whether I could balance everything—the studies are intense, and taking on more responsibility was a real challenge."*

At the same time, some viewed this challenge as an opportunity to develop time-management skills. Emily shared how the role taught her effective time management:

*"It was sometimes difficult to find time for studies, work, and mentoring tasks, but I learned a lot about effective time management."*

Another challenge was dealing with mentees who were unresponsive or disengaged. Emma described her experience with a mentee who rarely replied to messages, making it difficult to provide help:

*"One of my mentees rarely replied to messages, and although I wanted to help, I had to accept that he was going at his own pace."*

**Building Trust and Relationships**

Mentors emphasized the importance of establishing a safe and trusting relationship with their mentees, using personal experiences and active listening as strategies to strengthen these bonds. This relational focus was closely tied to their motivation to support and was seen as a critical factor in providing effective guidance. By sharing personal challenges, mentors created a sense of solidarity, making it easier for mentees to open up. Lisa explained how she shared her own insecurities and challenges to reduce her mentees’ feelings of isolation:

*"I shared my own insecurities and challenges with my mentees, which helped them feel less alone."*

Similarly, James noted that being open about his own experiences helped mentees trust him and share their own struggles:

*"Being open about my own experiences made it easier for mentees to trust me and share their own."*

Mentors also highlighted the importance of active listening and validating their mentees' feelings, as these approaches contributed to a supportive and trusting relationship. Emily described how listening without judgment and validating her mentees’ feelings was key to building strong relationships:

*"Listening without judgment and validating their feelings has been key to building strong relationships."*

Emma added that when mentees felt comfortable enough to share their problems, it was a sign of trust and that they felt seen:

*"When mentees felt comfortable enough to share their problems, it showed that they trusted me and felt seen."*

Consistency and reliability were also crucial for maintaining trust. Sarah prioritized being available and following through on commitments to ensure her mentees could rely on her:

*"I made it a priority to be available and always follow through so that my mentees knew they could rely on me."*

Olivia found that keeping promises and being present when her mentees needed her created a safe environment that encouraged openness:

*"By keeping my promises and being there when they needed me, I created a sense of safety that encouraged my mentees to open up."*

Through these strategies, mentors were able to build relationships characterized by trust and mutual respect, forming the foundation for meaningful interactions and effective support.

**Discussion**

This study highlights mentors' experiences in a student mentorship program, focusing on their motivations, skill development, challenges, and relational dynamics with mentees. The findings support and expand existing literature on student mentorship, illustrating how the mentor role contributes to both academic and personal growth for mentors. At the same time, the study emphasizes specific challenges related to balancing responsibilities and emotional demands in the mentor role.

A key finding is that mentors were motivated both by a desire to give back to the learning environment and by the opportunity for personal development. This aligns with previous research showing that student mentors often have dual motivations: a wish to help others and personal growth through the role (Terrion & Leonard, 2007). Our findings confirm this, as mentors described how their own experiences as new students made them aware of the need for support, motivating them to become a resource for new students facing similar challenges. Consistent with the literature (Colvin & Ashman, 2010), mentors also viewed the role as an opportunity to strengthen critical skills, including leadership and communication, which they perceive as relevant for future professional roles in healthcare. By participating as mentors, students saw the role as a practice ground for future nursing, applying and developing essential interpersonal skills such as empathy, active listening, and conflict resolution. These skills are particularly significant in healthcare professions, where communication and empathy are foundational (Buschlen & Johnson, 2014; Crisp & Cruz, 2009).

Regarding skill development, the results showed that mentors experienced significant growth, especially in communication, empathy, and emotional intelligence. This aligns with literature suggesting that mentorship fosters skills critical in healthcare professions, increasing social awareness and emotional competence (Rhodes & DuBois, 2008; Crisp & Cruz, 2009). Additionally, the study demonstrated that the mentor role boosted mentors' confidence and leadership abilities. This aligns with theories of professional identity development, where mentorship acts as a transitional role that prepares students for leadership responsibilities in their careers (Colvin & Ashman, 2010). By supporting students through academic and personal challenges, mentors practiced responsibility, decision-making, and facilitation of learning—skills that are valuable in both academic and clinical contexts.

However, mentors also faced numerous challenges, particularly in balancing mentoring duties with their studies and part-time work. The literature also supports that the mentor role can be time-consuming and mentally demanding, especially when combined with other academic commitments (Allen et al., 2004; Crisp & Cruz, 2009). Our study revealed that many mentors found it difficult to support mentees experiencing emotional challenges, such as anxiety and isolation, without being emotionally affected themselves. This underscores the need for mentors to receive training in setting boundaries and practicing self-care, which Terrion and Leonard (2007) also describe as essential for preventing emotional burnout among mentors.

**Conclusion**

This study has explored the experiences of student mentors in a nursing mentorship program, focusing on motivation, skill development, challenges, and relationship building. The findings provide valuable insights into the mentor role in nursing education, where both personal and professional development are central. At the same time, the results show that the mentor role can be demanding, requiring emotional resilience and the ability to balance responsibilities.

Mentors in this study reported developing key skills such as empathy, active listening, and leadership—skills directly relevant to future roles in healthcare. These findings extend previous literature by emphasizing how mentorship in healthcare education allows student mentors to practice skills that enhance their professionalism in interactions with patients and colleagues.

The study also offers concrete implications for designing mentorship programs, particularly the need for training and support. The findings highlight that educational institutions should include topics such as mental health and cultural competence in mentor training to address the diversity of student groups. Programs should also incorporate support mechanisms for mentors themselves to reduce emotional strain and enhance mentorship effectiveness. This is especially important in demanding educational pathways such as nursing, where mentors often encounter mentees with diverse challenges and complex needs.

- Allen, T. D., Eby, L. T., Poteet, M. L., Lentz, E., & Lima, L. (2004). Career benefits associated with mentoring for protégés: A meta-analysis. *Journal of Applied Psychology, 89*(1), 127–136. <https://doi.org/10.1037/0021-9010.89.1.127>
- Braun, V., & Clarke, V. (2006). Using thematic analysis in psychology. *Qualitative Research in Psychology, 3*(2), 77–101. <https://doi.org/10.1191/1478088706qp063oa>
- Braun, V., & Clarke, V. (2013). *Successful qualitative research: A practical guide for beginners.* SAGE Publications.
- Buschlen, E., & Johnson, M. (2014). The effects of an introductory leadership course on socially responsible leadership. *Journal of Leadership Education, 13*(1), 31–45. <https://doi.org/10.12806/V13/I1/R3>
- Colvin, J. W., & Ashman, M. (2010). Roles, risks, and benefits of peer mentoring relationships in higher education. *Mentoring & Tutoring: Partnership in Learning, 18*(2), 121–134. <https://doi.org/10.1080/13611261003678879>
- Crisp, G., & Cruz, I. (2009). Mentoring college students: A critical review of the literature between 1990 and 2007. *Research in Higher Education, 50*(6), 525–545. <https://doi.org/10.1007/s11162-009-9130-2>
- Kvale, S., & Brinkmann, S. (2015). *Det kvalitative forskningsintervju.* Gyldendal Norsk Forlag.
- NESH. (2016). *Forskningsetiske retningslinjer for samfunnsvitenskap, humaniora, juss og teologi.* Den nasjonale forskningsetiske komité for samfunnsvitenskap og humaniora (NESH). <https://www.etikkom.no/retningslinjer/>
- Nowell, L. S., Norris, J. M., White, D. E., & Moules, N. J. (2017). Thematic analysis: Striving to meet the trustworthiness criteria. *International Journal of Qualitative Methods, 16*(1), 1–13. <https://doi.org/10.1177/1609406917733847>
- Rhodes, J. E., & DuBois, D. L. (2008). Mentoring relationships and programs for youth. *Current Directions in Psychological Science, 17*(4), 254–258. <https://doi.org/10.1111/j.1467-8721.2008.00585.x>
- Terrion, J. L., & Leonard, D. (2007). A taxonomy of the characteristics of student peer mentors in higher education: Findings from a literature review. *Mentoring & Tutoring, 15*(2), 149–164. <https://doi.org/10.1080/13611260601086311>
