# Supplementary material for: Evaluating a Large Language Model’s Ability to Synthesize a Health Science Master’s Thesis: Case Study
Source: JMIR Form Res. 2025 Jul 3;9:e73248. doi: 10.2196/73248 (PMC12244274; doi:10.2196/73248)
Supplement: Multimedia Appendix 7 [file formative-v9-e73248-s007.docx]

**Associations Between Smoking, Alcohol Use, Physical Activity, Diet, and General Life Satisfaction: A Cross-Sectional Study**

Authored by ChatGPT4o based on synthetic data produced by ChatGPT-1o-preview and prompts from Pål Joranger and Asgeir Brevik

**Abstract**

**Background**
Lifestyle factors, including smoking, alcohol consumption, physical activity, and diet, are known to influence physical and mental health outcomes. However, their combined impact on general life satisfaction remains less explored.

**Objectives**
This study aimed to investigate the associations between lifestyle variables — smoking, alcohol use, physical activity, and fruit and vegetable consumption — and general life satisfaction among adults. Age, gender, and income were included as control variables.

**Methods**
A cross-sectional survey was conducted with 1,000 adult participants recruited via social media using a snowball sampling method. Data on lifestyle behaviors and life satisfaction were self-reported through standardized questionnaires. Multiple linear regression analysis was employed to evaluate the independent contributions of each lifestyle factor to life satisfaction, controlling for age, gender, and income. Spearman’s rank correlations were used to examine bivariate relationships.

**Results**
Higher fruit and vegetable consumption was significantly associated with increased life satisfaction (B=0.073, p<0.001). Alcohol use showed a small but significant negative association (B=−0.033, p=0.042). Age (B=0.009, p=0.023) and income (B=0.197, p<0.001) were positively related to life satisfaction, while women reported slightly higher satisfaction than men (B=0.215, p=0.011). Smoking and physical activity were not significant predictors in the multivariate model. The model explained approximately 9.9% of the variance in life satisfaction (R^2^=0.099).

**Conclusions**
The findings highlight the importance of healthy dietary habits, particularly fruit and vegetable consumption, and the negative implications of alcohol use for life satisfaction. Public health strategies should emphasize nutrition education and responsible alcohol consumption to enhance subjective well-being. Furthermore, policies aimed at improving socioeconomic conditions could positively influence life satisfaction. Future research should employ longitudinal designs to explore causal pathways between lifestyle behaviors and life satisfaction.

**Keywords:** Life satisfaction, lifestyle factors, smoking, alcohol, physical activity, diet, cross-sectional study.

**Introduction**

General well-being serves as a comprehensive indicator of quality of life and mental health, influencing both subjective experiences and objective outcomes, including physical health, social functioning, and economic stability (Diener et al., 2018). Understanding what contributes to life satisfaction has critical implications for public health efforts, as well-being is linked to a reduced risk of chronic illnesses, increased longevity, and higher productivity (Steptoe et al., 2015).

Lifestyle factors such as smoking, alcohol consumption, physical activity, and diet are well-documented in relation to physical health. Smoking, for instance, is strongly associated with a higher risk of heart disease and lung cancer (Gonzalez et al., 2018), and it has also been linked to poorer mental health and reduced life satisfaction (Strine et al., 2005). Alcohol consumption presents a more complex relationship with well-being; while moderate use can enhance social experiences, excessive consumption consistently leads to psychological distress and depression (Graham et al., 2007). Physical activity stands out as one of the most consistent predictors of improved mood and overall satisfaction, with well-established benefits for both mental and physical health (Penedo & Dahn, 2005; Warburton & Bredin, 2017). Similarly, diet—particularly one rich in fruits and vegetables—has been associated with better cognitive functioning and a lower risk of depression (Lai et al., 2014; Blanchflower et al., 2013).

Despite extensive research on the individual effects of these lifestyle factors, their combined impact on general well-being remains underexplored. Previous studies have often examined these factors in isolation, overlooking potential interactions between variables (e.g., Graham et al., 2007; Strine et al., 2005). This fragmented approach limits our understanding of how lifestyle choices collectively influence well-being. Moreover, there is a lack of research investigating how contextual and demographic factors, such as age, gender, and income, might moderate these relationships (Steptoe et al., 2015).

This study aims to address these gaps by examining the combined effects of smoking, alcohol consumption, physical activity, and diet on general well-being, while accounting for age, gender, and income. Adopting this holistic perspective provides deeper insights into how modifiable lifestyle factors influence subjective well-being, offering potential implications at both individual and societal levels.

**Method**

**Study Design and Participants**

This study utilized a cross-sectional design to investigate the associations between lifestyle variables (smoking, alcohol use, physical activity, and dietary habits) and general life satisfaction. Data collection was conducted using an online survey disseminated via social media platforms, including Facebook, Twitter, and Instagram. Recruitment followed a snowball sampling approach, where initial participants were encouraged to share the survey link with friends and acquaintances. This approach aimed to maximize reach and gather a diverse participant pool.

While snowball sampling effectively increased the sample size and geographic diversity, it introduced potential biases. These include overrepresentation of individuals who are more socially connected online and underrepresentation of those less active on social media (Baltar & Brunet, 2012). To mitigate these biases, we included questions on demographics and lifestyle to assess representativeness and conducted subgroup analyses to explore variability in responses.

**Eligibility Criteria**

Participants were eligible for inclusion if they (1) were aged 18 years or older, (2) had access to the internet and the ability to complete an online survey Or (3) provided informed consent.

Incomplete responses or entries that displayed clear patterns of invalidity (e.g., identical responses across all items) were excluded. This ensured the reliability and validity of the data collected.

**Data Collection**

The online survey was hosted on a secure platform to ensure participant privacy and confidentiality. Standardized items were included to measure the following variables (Table 1):

- **Life Satisfaction**: Measured with a single-item scale from 1 (low) to 10 (high), validated in previous research on subjective well-being (Diener et al., 1985).
- **Smoking and Alcohol Use**: Assessed via self-reported frequency on a 9-point Likert scale, ranging from 1 (never) to 9 (frequent use). These items align with validated instruments used in public health research (Strine et al., 2005).
- **Physical Activity (PhysActiv)**: Measured by frequency of engagement in physical activity on a scale from 1 (none) to 9 (daily). This scale has been used in studies linking exercise to mental health (Penedo & Dahn, 2005).
- **Dietary Habits (FiveADay)**: Measured as the average daily intake of fruits and vegetables on a scale from 1 (none) to 9 (5+ servings daily). This measure is consistent with dietary guidelines and research on nutrition and mental health (Blanchflower et al., 2013).

**Table 1**. The variables used.

| **Variable name** | **Label for the variables** | **The values ​​for the variables** |
| --- | --- | --- |
| Age | What is your age? | Number of years |
| Gender | Are you a woman or a man | Women = 1, Men = 0, 99 = missing |
| Education | What is your highest completed education? | 1=no education, 2=primary/primary school, 3=upper secondary school/upper secondary school/vocational school, 4 = College/university 3 years (bachelor's), 5= College/university 3 years+further education, 6= College/university 5 years (master's or more) |
| Income | What is the total annual gross income in the household? | 1=under 399000 NOK, 2=400000-599000 NOK, 3= 600000-799000 NOK, 4=800000-999000 NOK, 5=1000000-1199000 NOK, 6= 1200000NOK or more, 99 = missing |
| Smoking | Uses tobacco products such as smoking, snuff, e-cigarettes and other tobacco products | 1=never, 2=Less than 1 day, 3=1 day, 4=2 days, 5=3 days, 6=4 days, 7=5 days, 8=6 days, 9=7 days, 99 = missing |
| Alcohol | Consumes alcoholic beverages such as beer, wine, spirits, soft drinks etc. | 1=never, 2=Less than 1 day, 3=1 day, 4=2 days, 5=3 days, 6=4 days, 7=5 days, 8=6 days, 9=7 days, 99 = missing |
| PhysActiv | Exercising physical activity of high intensity for at least 15 minutes | 1=never, 2=Less than 1 day, 3=1 day, 4=2 days, 5=3 days, 6=4 days, 7=5 days, 8=6 days, 9=7 days, 99 = missing |
| FiveADay | Five units of vegetables or fruit per day | 1=never, 2=Less than 1 day, 3=1 day, 4=2 days, 5=3 days, 6=4 days, 7=5 days, 8=6 days, 9=7 days, 99 = missing |
| PerceivPhysHealth | Self-perceived physical health | Numbers between 1-10, where 1 means very bad and 10 means very good. |
| LifeSatisfaction | Satisfaction with life | Numbers between 1-10, where 1 means very dissatisfied and 10 means very satisfied. |

Demographic information, including age, gender, and income, was also collected. Gender was dichotomized as male (0) and female (1). Income was self-reported on a 10-point scale, where higher values indicated greater income levels.

**Statistical Methods**

*Descriptive Statistics:* Means, medians, standard deviations, and ranges were computed for continuous variables, while frequencies and percentages were calculated for categorical variables. These analyses provided an overview of the sample characteristics.

*Spearman’s Rank Correlation:* This non-parametric test was used to examine bivariate relationships between variables due to the ordinal nature of some measures. Spearman’s correlation is robust against non-normal distributions and is appropriate for ranking data (Hauke & Kossowski, 2011).

*Multiple Linear Regression:* Regression models were employed to evaluate the independent contributions of lifestyle variables and control variables (age, gender, and income) to life satisfaction.

To ensure the validity of the multiple regression analysis, key assumptions were tested. First, linearity was assessed using residual plots, which showed no significant deviations from the assumption. Second, homoscedasticity was tested using the Breusch-Pagan test, which indicated potential heteroscedasticity (p < 0.05). Third, multicollinearity was evaluated through Variance Inflation Factors (VIF), with all predictors showing acceptable values below 5, suggesting no significant multicollinearity.

Due to the violation of the homoscedasticity assumption, robust standard errors were applied to the regression analysis. This adjustment ensures reliable p-values and confidence intervals for the model coefficients, accounting for the presence of heteroscedasticity. The results from the robust regression analysis are presented in the results section.

**Ethical Considerations**

The study complied with ethical guidelines for research involving human participants. Informed consent was obtained from all respondents before participation. Participants were assured of their right to withdraw at any point without providing a reason. The study protocol was reviewed and approved by an institutional ethics review board, ensuring adherence to principles of confidentiality and data security.

**Strengths and Limitations**

The use of social media for recruitment allowed rapid and cost-effective data collection and access to a geographically diverse sample. However, this method introduced **self-selection bias** and limited generalizability due to the non-random sampling approach. Snowball sampling may have led to an overrepresentation of younger, digitally engaged individuals (Baltar & Brunet, 2012). To address these limitations, we included demographic controls in our analyses and reported subgroup differences to assess variability.

**Data Management and Analysis**

All data were anonymized prior to analysis to protect participants' identities. Statistical analyses were conducted using Python and SPSS, ensuring reproducibility and rigor. The results were reported in accordance with the STROBE checklist, ensuring transparency and clarity in the methodology and findings.

**Results**

**Descriptive Statistics**

The sample consisted of 1,000 participants, evenly distributed across gender (50% male and 50% female) and other demographic variables (Table 2). The mean life satisfaction score was 5.28 (SD = 1.36), with a range of 1.0 to 10.0. The median scores for smoking, alcohol consumption, physical activity, and fruit and vegetable intake (FiveADay) were all 5.0 on a scale from 1 to 9. Age ranged from 18 to 85 years, with a median of 50. Income followed a slightly skewed distribution, with a median category of 5, representing moderate income levels.

**Table 2** provides an overview of sample characteristics, presenting the central tendencies and variability for each variable.

| **Variable** | **Category** | **Number** | **Percent** |
| --- | --- | --- | --- |
| Age (mean, SD) |  | 1000 | 44.6 (13.0) |
| Gender | Menn | 480 | 48.0 |
|  | Kvinner | 520 | 52.0 |
| Income | 1 = under 399000 NOK | 100 | 10.0 |
|  | 2 = 400000-599000 NOK | 150 | 15.0 |
|  | 3 = 600000-799000 NOK | 200 | 20.0 |
|  | 4 = 800000-999000 NOK | 250 | 25.0 |
|  | 5 =1000000-1199000 NOK | 150 | 15.0 |
|  | 6 = 1200000 NOK or more | 150 | 15.0 |

**Correlation Analysis**

Spearman’s rank correlation analysis revealed several significant relationships between variables (Table 3).

- Life satisfaction was positively correlated with FiveADay (r=0.16, p<0.001), age (r=0.10, p=0.001), gender (r=0.10, p=0.002), and income (r=0.24, p<0.001).
- Negative correlations were found with smoking (r=−0.05, p=0.083) and alcohol consumption (r=−0.05, p=0.106), though these were not statistically significant.

Correlations among predictors included a strong positive relationship between FiveADay and physical activity (r=0.28, p<0.001) and a moderate association between smoking and alcohol consumption (r=0.20, p<0.001). Additionally, smoking was negatively correlated with income (r=−0.23, p<0.001), suggesting that higher income levels are associated with reduced smoking prevalence.

**Table 3** lists all pairwise correlations and their respective significance levels.

|  | **LifeSatis-faction** | **Smoking** | **Alcohol** | **PhysActiv** | **FiveADay** | **Age** | **Gender** | **Income** |
| --- | --- | --- | --- | --- | --- | --- | --- | --- |
| **LifeSatisfaction** | 1.00 | -0.05 (p=0.083) | -0.05 (p=0.106) | -0.02 (p=0.448) | 0.16 (p<0.001) | 0.10 (p=0.001) | 0.10 (p=0.002) | 0.24 (p<0.001) |
| **Smoking** |  | 1.00 | 0.20 (p<0.001) | 0.22 (p=0.121) | -0.13 (p<0.001) | -0.10 (p=0.001) | 0.03 (p=0.299) | -0.23 (p<0.001) |
| **Alcohol** |  |  | 1.00 | 0.05 (p=0.546) | -0.13 (p<0.001) | 0.02 (p=0.812) | 0.03 (p=0.431) | -0.10 (p=0.097) |
| **PhysActiv** |  |  |  | 1.00 | 0.28 (p<0.001) | 0.25 (p<0.001) | 0.03 (p=0.597) | 0.00 (p=0.973) |
| **FiveADay** |  |  |  |  | 1.00 | 0.25 (p<0.001) | -0.13 (p<0.001) | 0.09 (p=0.101) |
| **Age** |  |  |  |  |  | 1.00 | 0.01 (p=0.663) | 0.01 (p=0.863) |
| **Gender** |  |  |  |  |  |  | 1.00 | 0.01 (p=0.780) |
| **Income** |  |  |  |  |  |  |  | 1.00 |

**Multivariable Regression Analysis**

Multiple linear regression was conducted to evaluate the independent contributions of lifestyle and demographic factors to life satisfaction (Table 4). The model explained 9.9% of the variance in life satisfaction (R^2^=0.099, p<0.001).

Key findings included:

- **FiveADay**: A significant positive predictor (B=0.073, p<0.001). A one-unit increase in servings of fruits and vegetables was associated with a 0.073-point increase in life satisfaction.
- **Alcohol Consumption**: A small but significant negative association (B=−0.033, p=0.042).
- **Age**: Positively associated with life satisfaction (B=0.009, p=0.023).
- **Gender**: Women reported slightly higher satisfaction levels than men (B=0.215, p=0.011).
- **Income**: A strong positive predictor (B=0.197, p<0.001).

Smoking and physical activity were not statistically significant predictors, although smoking showed a trend toward a small negative association (B=−0.026, p=0.124).

**Table 4** presents regression coefficients, confidence intervals, and significance levels. It provides a comprehensive overview of each variable's contribution to life satisfaction.

| **Variable** | **B** | **p-value** | **95% CI Lower** | **95% CI Upper** |
| --- | --- | --- | --- | --- |
| const | 3.860 | <0.001 | 3.354 | 4.365 |
| Smoking | 0.026 | 0.124 | -0.007 | 0.060 |
| Alcohol | -0.033 | 0.042 | -0.065 | -0.001 |
| PhysActiv | -0.028 | 0.093 | -0.060 | -0.005 |
| FiveADay | 0.073 | <0.001 | 0.039 | 0.107 |
| Age | 0.009 | 0.023 | 0.001 | 0.017 |
| Gender | 0.215 | 0.011 | 0.050 | 0.381 |
| Income | 0.197 | <0.001 | 0.149 | 0.246 |

**Discussion**

This study examined how lifestyle factors such as smoking, alcohol consumption, physical activity, and diet correlate with overall well-being. The results indicated that a higher intake of fruits and vegetables (FiveADay) was significantly associated with increased life satisfaction, while alcohol consumption showed a small but significant negative relationship. Age and income were also positively associated with well-being, whereas smoking and physical activity were not significant predictors in the multivariate model. These findings underscore the importance of diet and financial stability for subjective well-being and highlight the need for further research on how lifestyle factors influence well-being.

**Methodological Discussion**

One of the key strengths of this study is its relatively large sample size (N = 1000), which provides sufficient statistical power to detect subtle effects of lifestyle factors on well-being. Additionally, the simultaneous evaluation of multiple factors enabled a more comprehensive analysis of their relative contributions, addressing a gap in the literature where lifestyle factors are often studied in isolation (Blanchflower et al., 2013; Graham et al., 2007). The use of a standardized survey platform also ensured structured data collection, facilitating replication.

Despite these strengths, the study has several limitations that warrant consideration.

First, the recruitment method, using social media and the snowball sampling technique, introduces selection bias. While this approach effectively increased reach and sample size, it likely resulted in a participant pool that was younger, more tech-savvy, and had greater access to health resources compared to the general population (Baltar & Brunet, 2012). This limits the generalizability of the findings. Future studies should consider more representative recruitment methods, such as stratified random sampling, to enhance the transferability of results.

Second, all variables were measured through self-reported data, which can introduce information bias due to social desirability or inaccurate reporting (Althubaiti, 2016). For instance, participants might underreport alcohol consumption or overestimate their daily intake of fruits and vegetables. To mitigate this, future research should incorporate objective measures, such as biomarkers for nutrition or activity trackers for physical activity, to improve the accuracy and reliability of findings.

Third, the cross-sectional design of the study precludes the establishment of causal relationships between lifestyle factors and well-being. It is plausible that individuals with high well-being are more inclined to adopt healthy lifestyle habits, rather than lifestyle factors directly influencing well-being (Levin, 2006). Longitudinal studies are needed to investigate causality and better understand the dynamic interactions between lifestyle factors and subjective well-being.

Lastly, while age, gender, and income were included as control variables, other critical factors such as mental health, social networks, and genetic predispositions were not accounted for. These factors may have influenced the results and should be included in future analyses to provide a more comprehensive understanding (Weston et al., 2019).

To build on the findings of this study, future research should consider employing longitudinal designs to explore the causal mechanisms linking lifestyle factors and life satisfaction (Steptoe et al., 2015). Incorporating objective measurements of lifestyle factors, such as biomarkers for diet or alcohol consumption and activity monitors for physical activity, would reduce information bias and enhance data accuracy. Furthermore, future studies should examine the interactions between lifestyle factors and other variables, including social determinants like access to healthcare, to identify complex relationships that influence well-being.

**Discussion of Results**

The findings from this study shed light on the relationships between lifestyle factors and life satisfaction, with several key implications for public health and future research.

A positive association was observed between the intake of fruits and vegetables (FiveADay) and life satisfaction, consistent with previous studies highlighting the role of nutrition in mental health. For instance, Blanchflower et al. (2013) found a correlation between higher fruit and vegetable consumption and improved quality of life, while Lai et al. (2014) reported a significant link between a healthy diet and lower depression rates. The psychological benefits of nutrition may partly be attributed to the physiological effects of nutrients on brain function, such as reduced inflammation and increased production of neurotransmitters like serotonin. These findings emphasize the importance of promoting healthy dietary habits as part of public health strategies. Interventions such as subsidizing fruits and vegetables and educational campaigns on nutrition’s impact on mental health could play a pivotal role.

Alcohol consumption, on the other hand, showed a small but significant negative association with life satisfaction, supporting previous research by Graham et al. (2007), which documented an increased risk of depression with higher alcohol use. While moderate consumption is sometimes associated with social well-being, excessive intake can lead to cumulative health risks and social challenges that negatively affect well-being. These results underline the need for targeted alcohol prevention measures, particularly for high-risk groups. Interventions promoting responsible drinking could improve life satisfaction at the population level.

In contrast to some earlier studies that highlighted the positive effects of physical activity on mental health (Penedo & Dahn, 2005), no significant association was found between physical activity and life satisfaction in this study. This discrepancy may be explained by moderating factors such as social support or individual differences in exercise habits. Warburton and Bredin (2017) suggest that the quality of physical activity, including factors like exercise intensity and social interaction, may be crucial to its impact on well-being. Future interventions should explore how different types of physical activity and social contexts influence life satisfaction, with group activities or those fostering social connections potentially yielding greater benefits than solitary exercise.

Although no significant relationship was identified between smoking and life satisfaction in this study, prior research, such as Strine et al. (2005), has indicated that smoking can reduce subjective well-being. One possible explanation is that smokers in this sample may have already internalized the negative health consequences of smoking, diminishing its observable effect on well-being. Tobacco prevention measures, including cessation support programs, could potentially enhance both physical and mental health, particularly for individuals with lower well-being.

The positive associations between age and income with life satisfaction align with prior research. Studies such as Blanchflower and Oswald (2008) report increasing satisfaction with age, while Diener et al. (2010) emphasize the importance of economic stability for well-being. Economic security may facilitate access to better health care, education, and social opportunities, while older individuals may benefit from enhanced coping skills and greater adaptation to life’s challenges. Policymakers could focus on economic support and education programs for low-income groups to significantly impact their subjective well-being. For older populations, initiatives promoting social participation and belonging could further enhance life satisfaction.

Finally, women in this study reported higher life satisfaction than men, a finding consistent with some previous research (Pinquart & Sörensen, 2001). However, other studies, such as Alesina et al. (2004), reveal mixed results, suggesting that social expectations and gender roles may influence well-being reporting. Interventions that account for gender differences, including tailored health and well-being programs for men and women, could help achieve more balanced outcomes in subjective well-being.

Overall, these results provide important insights into the complex interplay of lifestyle factors and well-being, offering guidance for both policy and future research directions.

**Future Research**

Future studies should employ longitudinal designs to explore the causal mechanisms between lifestyle factors and well-being. This would provide a deeper understanding of the direction of these relationships—for instance, whether higher well-being leads to healthier lifestyle choices, or vice versa. Furthermore, objective measurements of lifestyle behaviors, such as biomarkers for diet and activity trackers for physical activity, should be included to reduce information bias. It would also be valuable to investigate how social determinants, such as access to healthcare and social support, moderate these relationships.

**Policy Relevance**

The findings have significant implications for policy strategies aimed at promoting mental health and well-being. Interventions such as subsidizing healthy food and educational campaigns to raise awareness about the importance of nutrition for well-being could be effective. Additionally, the findings highlight the need for alcohol prevention initiatives that encourage responsible consumption, particularly among high-risk groups. Policies that enhance economic stability, such as job creation and financial support programs, could also improve subjective well-being, especially among low-income populations.

**Conclusion**

This study highlights the influence of lifestyle factors and sociodemographic conditions on overall well-being in adults. Higher fruit and vegetable intake was associated with increased life satisfaction, while alcohol consumption had a negative impact. Age and income emerged as strong predictors of well-being, whereas smoking and physical activity showed no significant associations. Future research should utilize longitudinal designs and objective measures to clarify causal mechanisms and minimize bias. These findings underscore the need for policy interventions such as subsidizing healthy food, alcohol prevention initiatives, and economic support programs to enhance population well-being.

**References**

Alesina, A., Di Tella, R., & MacCulloch, R. (2004). Inequality and happiness: Are Europeans and Americans different? *Journal of Public Economics, 88*(9-10), 2009–2042. <https://doi.org/10.1016/j.jpubeco.2003.07.006>

Althubaiti, A. (2016). Information bias in health research: Definition, pitfalls, and adjustment methods. *Journal of Multidisciplinary Healthcare, 9*, 211–217. <https://doi.org/10.2147/JMDH.S104807>

Baltar, F., & Brunet, I. (2012). Social research 2.0: Virtual snowball sampling method using Facebook. *Internet Research, 22*(1), 57–74. <https://doi.org/10.1108/10662241211199960>

Blanchflower, D. G., & Oswald, A. J. (2008). Is well-being U-shaped over the life cycle? *Social Science & Medicine, 66*(8), 1733–1749. <https://doi.org/10.1016/j.socscimed.2008.01.030>

Blanchflower, D. G., Oswald, A. J., & Stewart-Brown, S. (2013). Is psychological well-being linked to the consumption of fruit and vegetables? *Social Indicators Research, 114*(3), 785–801. <https://doi.org/10.1007/s11205-012-0173-y>

Craney, T. A., & Surles, J. G. (2002). Model-dependent variance inflation factor cutoff values. *Quality Engineering, 14*(3), 391–403. <https://doi.org/10.1081/QEN-120001878>

Diener, E., Emmons, R. A., Larsen, R. J., & Griffin, S. (1985). The Satisfaction with Life Scale. *Journal of Personality Assessment, 49*(1), 71–75. <https://doi.org/10.1207/s15327752jpa4901_13>

Diener, E., Kahneman, D., Tov, W., & Arora, R. (2010). Income's association with judgments of life versus feelings. In E. Diener, J. F. Helliwell, & D. Kahneman (Eds.), *International differences in well-being* (pp. 3–15). Oxford University Press. <https://doi.org/10.1093/acprof:oso/9780199732739.003.0001>

Diener, E., Oishi, S., & Tay, L. (2018). Advances in subjective well-being research. *Nature Human Behaviour, 2*(4), 253–260. <https://doi.org/10.1038/s41562-018-0307-6>

Graham, K., Massak, A., Demers, A., & Rehm, J. (2007). Does the association between alcohol consumption and depression depend on how they are measured? *Alcoholism: Clinical and Experimental Research, 31*(1), 78–88. <https://doi.org/10.1111/j.1530-0277.2006.00274.x>

Hauke, J., & Kossowski, T. (2011). Comparison of values of Pearson’s and Spearman’s correlation coefficients on the same sets of data. *Quaestiones Geographicae, 30*(2), 87–93. <https://doi.org/10.2478/v10117-011-0021-1>

Lai, J. S., Hiles, S., Bisquera, A., Hure, A. J., McEvoy, M., & Attia, J. (2014). A systematic review and meta-analysis of dietary patterns and depression in community-dwelling adults. *The American Journal of Clinical Nutrition, 99*(1), 181–197. <https://doi.org/10.3945/ajcn.113.069880>

Levin, K. A. (2006). Study design III: Cross-sectional studies. *Evidence-Based Dentistry, 7*(1), 24–25. <https://doi.org/10.1038/sj.ebd.6400375>

Penedo, F. J., & Dahn, J. R. (2005). Exercise and well-being: A review of mental and physical health benefits associated with physical activity. *Current Opinion in Psychiatry, 18*(2), 189–193. <https://doi.org/10.1097/00001504-200503000-00013>

Steptoe, A., Deaton, A., & Stone, A. A. (2015). Subjective wellbeing, health, and ageing. *The Lancet, 385*(9968), 640–648. <https://doi.org/10.1016/S0140-6736(13)61489-0>

Strine, T. W., Chapman, D. P., Balluz, L., Moriarty, D. G., & Mokdad, A. H. (2005). The associations between life satisfaction and health-related quality of life, chronic illness, and health behaviors among U.S. community-dwelling adults. *Journal of Community Health, 30*(4), 299–305. DOI: 10.1007/s10900-007-9066-4

Warburton, D. E., & Bredin, S. S. (2017). Health benefits of physical activity: A systematic review of current systematic reviews. *Current Opinion in Cardiology, 32*(5), 541–556. <https://doi.org/10.1097/HCO.0000000000000437>

Weston, S. J., Hill, P. L., & Jackson, J. J. (2014). Personality traits predict the onset of disease. *Social Psychological and Personality Science, 10*(2), 225–233. <https://doi.org/10.1177/1948550614553348>
