# Supplementary material for: Evaluating a Large Language Model’s Ability to Synthesize a Health Science Master’s Thesis: Case Study
Source: JMIR Form Res. 2025 Jul 3;9:e73248. doi: 10.2196/73248 (PMC12244274; doi:10.2196/73248)

Spearman Rank Correlation used to examine bivariate relationships using SPSS to check the analyses performed by ChatGPT 4.


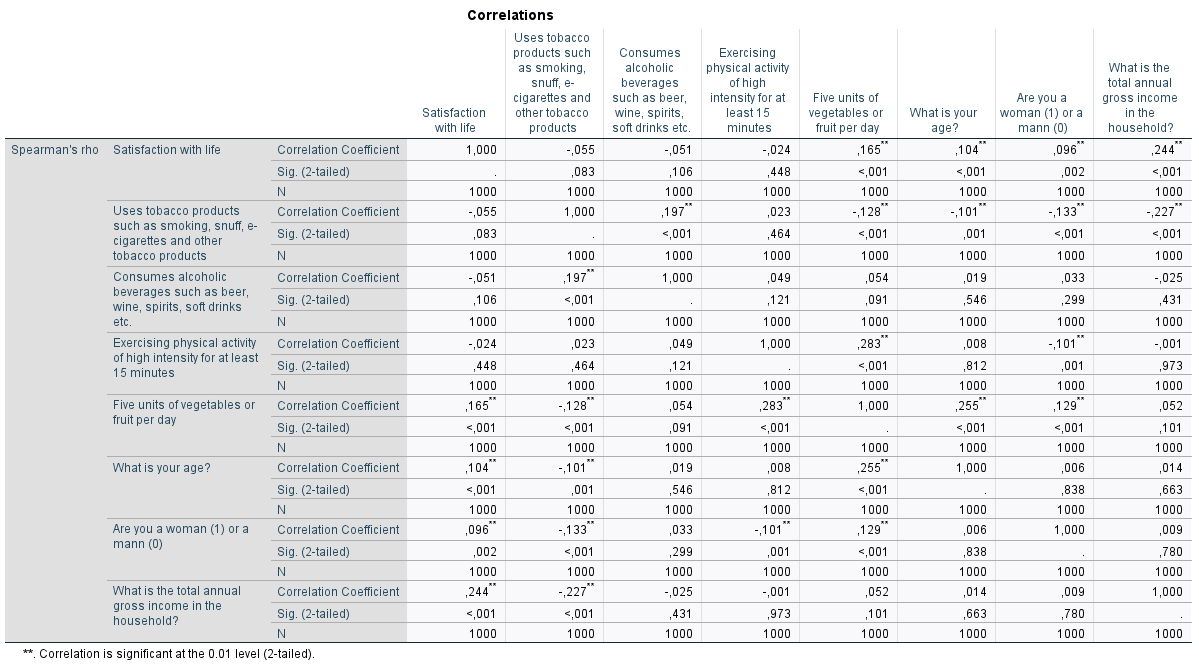

Supplement: Multimedia Appendix 8 [file formative-v9-e73248-s008.docx]
